# Dynamic and stable hippocampal representations of social identity and reward expectation support associative social memory in male mice

**DOI:** 10.1038/s41467-023-38338-3

**Published:** 2023-05-05

**Authors:** Eunji Kong, Kyu-Hee Lee, Jongrok Do, Pilhan Kim, Doyun Lee

**Affiliations:** 1 Center for Cognition and Sociality, Institute for Basic Science, Daejeon, 34126 Republic of Korea; 2 Graduate School of Medical Science and Engineering, Korea Advanced Institute of Science and Technology, Daejeon, 34141 Republic of Korea; 3 KI for Health Science and Technology (KIHST), Korea Advanced Institute of Science and Technology, Daejeon, 34141 Republic of Korea; 4 Department of Biological Sciences, Korea Advanced Institute of Science and Technology, Daejeon, 34141 Republic of Korea

**Keywords:** Social behaviour, Agency, Hippocampus, Reward

## Abstract

Recognizing an individual and retrieving and updating the value information assigned to the individual are fundamental abilities for establishing social relationships. To understand the neural mechanisms underlying the association between social identity and reward value, we developed Go-NoGo social discrimination paradigms that required male subject mice to distinguish between familiar mice based on their individually unique characteristics and associate them with reward availability. We found that mice could discriminate individual conspecifics through a brief nose-to-nose investigation, and this ability depended on the dorsal hippocampus. Two-photon calcium imaging revealed that dorsal CA1 hippocampal neurons represented reward expectation during social, but not non-social tasks, and these activities were maintained over days regardless of the identity of the associated mouse. Furthermore, a dynamically changing subset of hippocampal CA1 neurons discriminated between individual mice with high accuracy. Our findings suggest that the neuronal activities in CA1 provide possible neural substrates for associative social memory.

## Abstract (web summary)

The ability to recognize an individual and retrieve related information is crucial for social animals. Here, the authors employ a new social recognition paradigm to show that dorsal CA1 neurons distinguish individual mice and encode associated reward information.

## Introduction

Within a social group, animals frequently and repeatedly interact with each other. During these interactions, animals recognize their social counterparts as unique individuals, retrieve the information related to the individual that has been accumulated through previous interactions, and incorporate the new information obtained in the current interaction. These abilities are essential for adaptive social behaviors in cohesive social groups^1,2^. In particular, associating an individual with a subjective value regarding the social experiences with the individual and updating that value is critical for establishing social relationships^3^. For example, evaluating how pleasant and rewarding it was to interact with an individual is essential for building friendship.

The hippocampus has long been considered as an essential brain area for processing episodic information encompassing where, when, and what components of daily experiences^4,5^. Establishing associations between these episodic components in a correct sequence is believed to be essential for representing episodes. In addition, several studies demonstrated that the hippocampus is also important for processing social information, which is another important episodic component^6–13^.

Another set of studies demonstrated that reward-related information is also encoded in the hippocampus, especially in the CA1 subregions. During spatial navigation in an environment in which reward is provided at a certain location, hippocampal neurons overly represent the reward location^14–16^. In addition, altering reward locations strongly modulates the spatial firing of CA1 neurons^17,18^. Moreover, hippocampal CA1 neurons encode the expectation of reward at a certain location during navigation^19^. A recent study further demonstrated that CA1 neurons encode reward expectation regardless of the location of the reward, as well as spatial context, suggesting the presence of a dedicated neuronal population for reward coding^20^. In addition, CA1 neurons were shown to represent the quantitative estimate of the expected reward^21^. Furthermore, the CA1, but not other hippocampal subregions, appeared to be critical for updating the reward value^22^. Finally, recent studies demonstrated that CA1 neurons can encode an abstract space in which one or two axes represent reward value^23,24^.

Although reward value processing in the rodent hippocampus is mostly found in the context of spatial processing, there are observations that the hippocampus is required for associating reward with social information. In the social transmission of food preference paradigms, rodents were shown to remember the association between the food scent and the carbon disulfide sent in the breath of a demonstrator that had recently eaten the scented food. The hippocampus appeared to be important for the ability^25–28^ (but also see Burton et al.^29^). Furthermore, one study demonstrated that golden hamsters are able to discriminate the familiar conspecific with which they had frightening experiences from the one with which they interacted neutrally^7^. The inactivation of the dorsal CA1 region impaired this ability, indicating that this area is critical for associating social identities with a negative value. In the human hippocampus, affiliation to others and the difference in power between self and others are represented by hippocampal activity, thus emphasizing the role of the hippocampus in associating value and social information^3^.

Considering the wealth of evidence of reward processing in the dorsal CA1 and the fact that the dorsal CA1 is heavily projected from the neighboring dorsal CA2, which is an essential brain area for social information processing^8,30–32^, the dorsal CA1 is a strong candidate region where the association between social identities and reward value might occur. Therefore, we aimed to identify neural activities related to individual identity as well as associated reward values in the dorsal CA1 hippocampus in this study. To do so, we developed novel Go-NoGo individual discrimination paradigms, in which subject mice were required to associate each stimulus mice with the presence or absence of rewards. The stimulus mice used in these paradigms were male littermates, and were thus identical in sex, age, and genetic makeup, and were also equally familiar to the subject mice. Therefore, discrimination between these stimulus mice should be made solely based on their individually unique characteristics, rather than on a difference in the social categories of the stimulus mice. Using these paradigms, we showed that mice discriminate between individual stimulus mice via a brief nose-to-nose investigation, and that long-term memories of the stimulus mice were maintained in the subject mice that were not socially housed. We also found that dorsal hippocampal activities were necessary for discriminating between individual conspecifics, but not between non-social odors. We further demonstrated that neural activity in the dorsal CA1 hippocampus provides a stable representation of reward expectations associated with stimulus mice but not non-social odors. These representations of the reward expectation were independent of the identity of the associated stimulus mouse. In addition, we demonstrated that social identities were encoded with high accuracy by dynamic neuronal populations in the dorsal CA1.

## Results

### Individual discrimination paradigm

**Fig. 1:**
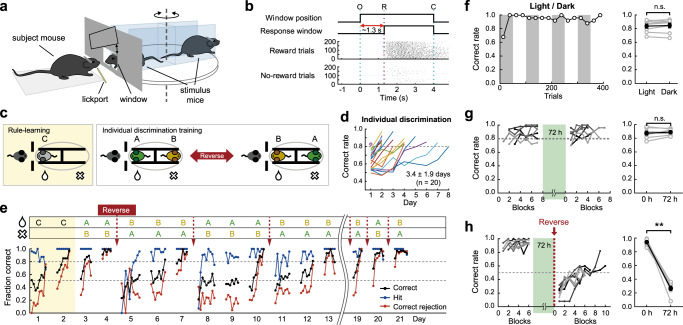
Discrimination of individual conspecific mice. **a** Schematic of the behavioral setup. **b** Task structure. O, window opening. R, start of response window. C, window closing (*top*). Raster plots of licking responses of a well-trained mouse in reward (*middle*) and no-reward (*bottom*) trials. **c** Schematics of rule-learning step (discrimination between a mouse and an empty head-fixing device; *left*) and individual discrimination task (*right*). Stimulus-reward contingency was reversed if necessary. A, B, and C indicate different stimulus mice. **d** Time courses for individual discrimination training. The correct rates for the individual discrimination task were plotted until they reached above 80%. The purple circle indicates that the mouse reached an above 80% correct rate in one session. **e** Learning curves of an example mouse that went through repeated reversal learning. Each filled circle represents the mean performance for fifty trials. **f** Left: Behavioral performance under an alternating lighting condition in an example session. Gray shades indicate dark periods. The correct rate was calculated for every 25 trials. Right: Group comparison in mean correct rates between light and dark conditions (two-sided Wilcoxon signed-rank test; *p* = 0.383, *n* = 8 mice). n.s., not significant. **g** Left: behavioral performance of each subject mouse before and after a 72 h break. The green shade indicates a 72 h break period during which the subject mice were single-housed in their home cages. There were 50 trials for each block. Each level of gray indicates results from each subject mouse. Right: Group comparison of mean correct rates (two-sided Wilcoxon signed-rank test, *p* = 0.688, *n* = 7). **h** Left: Similar to **g**, but reward contingency was reversed after the 72 h break. Right: Group comparison of the mean correct rates over 100 trials before and after the break (two-sided Wilcoxon signed-rank test, *p* = 0.008, *n* = 8). Gray circles, different mice. Black circles, mean across mice. Error bars, SEM across mice. n.s., not significant. Source data are provided as a Source Data file.

We developed an individual discrimination paradigm in which subject mice learned to discriminate between two familiar male littermate mice to obtain a water reward (Fig. 1a and Supplementary Fig. 1; see Methods). The two stimulus mice were head-fixed on the opposite side of a rotating platform and presented through an interaction window in random order. One stimulus mouse was associated with a reward, whereas the other was not. After a 0.8‒1.3 s grace period from the opening of the interaction window, licking the lickport triggered a water reward when the reward-associated mouse was presented, but not when the no-reward-associated one was presented (Go-NoGo task; Fig. 1b). To facilitate learning, the subject mice were first subjected to rule learning, in which they learned to distinguish between a stimulus mouse and an empty head-fixing device (Fig. 1c, left). The majority of the subject mice reached a success rate of approximately 80% within 4 days (3.0 ± 0.9 days, 20 mice). The subject mice then proceeded to the individual discrimination phase (Fig. 1c, right). The subject became familiarized with the initially novel stimulus mice only during the task. The subject mice performed ~300 trials in a daily session and reached a > 80% success rate in 3–4 days (3.4 ± 1.9 days, 20 mice) (Fig. 1d). After the initial training, if necessary, the mice were further trained under the reversed reward contingency, in which the previously reward-associated mouse was not associated with a reward and the no-reward-associated mouse was reward-associated (Fig. 1c, e).

Although the individual discrimination tasks were conducted under dim light conditions, we could not exclude the possibility that these mice performed the task relying at least partially on visual information. Thus, we tested a group of well-trained subject mice under alternating lighting conditions to examine whether visual information was necessary for discriminating between individual mice. Behavioral performance did not differ between the light and dark conditions, suggesting that visual information was not required for distinguishing between individual mice (Fig. 1f).

### Long-lasting memories of individual mice

Rodents display a natural preference for interacting more with a novel than a familiar conspecific. Therefore, if rodents are repeatedly exposed to the same conspecifics, the investigation time decreases in the later interactions compared with the first encounter. The maintenance of the memory of previously encountered conspecifics has been inferred from the decreased investigation^6,33–35^. While social memory tested with the social novelty preference paradigms appeared to last briefly (~1 h)^33,36,37^, the duration of social memory in mice has been shown to depend on housing conditions. Long-term social memory lasting more than 24 h is observed only in group-housed mice^6,38^. If a mouse is housed alone for more than 24 h, it does not show a decreased investigation of the previously exposed conspecifics. This phenomenon is interpreted as a failure to remember the familiar conspecific^6^. However, the comparison of the time spent investigating a novel and familiar conspecific is an indirect measure of social memory. It is also possible that lack of social interaction in the home cage increases motivation for social investigation, leading to equal investigation of both familiar and novel conspecifics. Interestingly, long-lasting social memory in individually housed rodents has been reported in a social memory paradigm that does not rely on the preference for social novelty^7^. Because the subject mice used in the current study were all individually housed, we directly investigated the duration of the memory of individual conspecifics in individually housed mice.

Using a group of well-trained mice, we compared task performance before and after a 72 h break. We observed that the subject mice were able to discriminate between two stimulus mice at a high rate from the beginning of the session after the 72 h break (Fig. 1g). This finding suggests that individually housed mice can hold social memories for 72 h. To exclude the possibility that such high performance at the beginning of the session was caused by rapid re-learning, rather than long-lasting social memory, we further tested a separate group of mice under a reversed rule after a 72 h break. We found that task performance at the beginning of the session decreased to a level of <50%, indicating that the subject mice performed the task according to the previous mouse-reward contingency before the break (Fig. 1h). Taken together, these results demonstrate that mice could hold memories of individual conspecifics for at least 72 h, even though they were individually housed. These findings are in sharp contrast with previous observations that long-term social memory is maintained only in group-housed rodents^6,33,38^.

### Individual discrimination requires the dorsal CA1 hippocampus

**Fig. 2:**
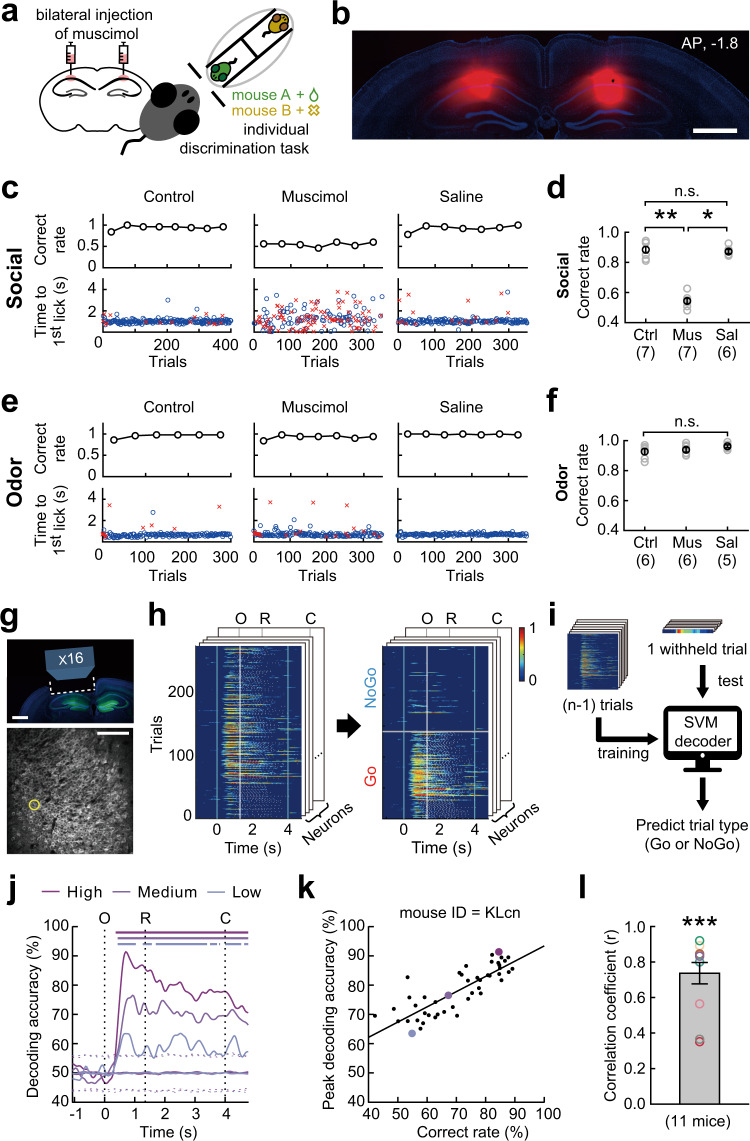
Individual discrimination requires the dorsal CA1 hippocampus. **a** Experimental scheme. **b** Spread of muscimol in the dorsal hippocampus. Red, BODIPY-conjugated muscimol. Blue, DAPI. Scale bar, 1 mm. See Supplementary Fig. 2 for the images for the other eight mice. **c** Muscimol, but not saline injection decreased the behavioral performance and dispersed the response time. Blue circles and red crosses indicate the response time for correct and incorrect licks, respectively. **d** Group comparison (Kruskal Wallis test with post hoc Dunn’s multiple comparisons: Ctrl vs. Mus, *p* = 0.003; Mus vs. Sal, *p* = 0.014). Ctrl, no injection; Mus, muscimol; Sal, saline. Numbers below the labels indicate the numbers of mice. **e** Muscimol injection had no effect on the non-social odor discrimination task (citral and 1-butanol). The example behavioral data in **c** and **e** were obtained from the same mouse. **f** Group comparison (Kruskal–Wallis test). n.s., not significant. **g** Top: Schematic of imaging window. Bottom: GCaMP6f signals in dorsal CA1. **h** Normalized changes in calcium signal during individual discrimination. Trials were sorted according to the trial type on the right. O, window opening. R, start of response window. C, window closing. The activity heat map on the front was obtained from the neuron marked with the yellow circle in **g**. **i** Support vector machine (SVM) decoding procedure. **j** SVM decoding accuracies at different behavioral performance levels in an example mouse (KLcn mouse in Supplementary Fig. 5). Sessions for the top, middle, and bottom performance level are indicated in **k** with matched color. Upper bars indicate the period of significant decoding (shuffling test, two-side, *p* < 0.05). Dashed lines represent top and bottom 2.5% of decoding accuracies obtained from shuffled data. **k** Peak decoding accuracy correlated with behavioral performance (Pearson’s *r* = 0.82, *p* = 7.87 × 10^−13^). Each dot indicates each behavioral session. The black line indicates the regression line. **l** Distribution of correlation coefficients for each mouse (mean ± SEM, two-sided paired t-test, *p* = 3.78 × 10^−7^). Source data are provided as a Source Data file.

The rodent hippocampus has been suggested as a critical structure for social information processing and memories^6–13,30–32,39–41^. In this study, bilateral injection of muscimol into the area centered on the dorsal CA1 hippocampus of well-trained mice (seven mice) decreased the behavioral performance to the near chance level (Fig. 2a–d, Supplementary Fig. 2). In addition, it dispersed the response time (time to the first lick in each trial), which was otherwise near the start of the response window. However, the injection of saline into the same brain area did not alter the behavioral performance or response time (Fig. 2c, d). To examine whether the effect of inactivating the dorsal hippocampus was specific to individual discrimination, we trained a set of mice (six mice; four of which were tested in both individual and non-social odor discrimination tasks) on a non-social odor discrimination task, which was identical to the individual discrimination task, with the exception that two non-social odors (citral and 1-butanol) were used as stimuli. We found that muscimol injection into the same brain area did not affect the ability of mice to discriminate between non-social odors and the response time (Fig. 2e, f). These results demonstrate that the dorsal hippocampus plays a necessary role in discriminating individual conspecifics, rather than a general role in discriminating odors or in encoding the task structure.

Next, we monitored the activity of dorsal hippocampal CA1 pyramidal neurons in 11 Thy1-GCaMP6f transgenic mice^16,42^ using two-photon calcium imaging (Fig. 2g and Supplementary Fig. 3), while performing the individual discrimination task. Typically, calcium imaging started once the behavioral performance reached near the high-performance criterion and continued throughout most of sessions while the subject mice were experiencing multiple events of reversal learning (Supplementary Fig. 4). Therefore, CA1 neuronal activity was monitored at the various levels of behavioral performance. We examined whether the hippocampal neuronal activity provides sufficient information for decoding trial types (Go and NoGo trials) using a support vector machine (SVM; Fig. 2h, i). The SVM decoding accuracy was near the chance level until ~0.3 s after the window opened, and rapidly increased thereafter (Fig. 2j). The peak decoding accuracy also increased according to the behavioral performance (Fig. 2j, k). A significant correlation between decoding accuracy and behavioral performance was observed in many of these mice (eight out of 11 mice, *p* < 0.05; Supplementary Fig. 5), as well as in the pooled data set (Fig. 2l).

Taken together, these findings suggest that dorsal CA1 hippocampal neurons provide critical information for discriminating individual conspecifics.

### Hippocampal CA1 neurons represent the expectation of a reward

Next, we examined the nature of the information encoded by the hippocampal activity. In the Go-NoGo task, recognition of the stimulus mouse is immediately followed by the prediction of the presence or absence of a reward. Therefore, it is unclear what information is represented by the neural activity that selectively responds in Go or NoGo trials. One possible strategy to overcome this ambiguity is to monitor the activity of the same neurons while reversing the stimulus-reward contingency and testing whether the neuronal activity follows stimuli or reward prediction. Among the 11 mice, six learned reversed rule at least once. After reversing the reward contingency, mice were considered to have learned and performed the task according to the reversed rule if the mice reached a high behavioral performance criterion (both hit and correct rejection rates > 80%) and the high-performance period contained more than 200 trials. After experiencing several rounds of reversal learning, five of them could learn it in a day. In three mice, reversal learning could be done repeatedly for four consecutive days (Supplementary Fig. 4).

**Fig. 3:**
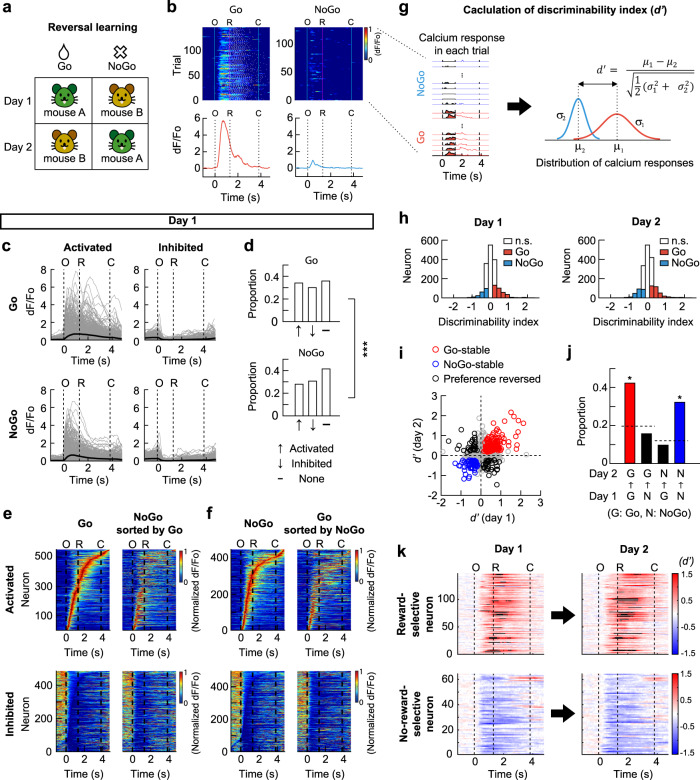
Hippocampal CA1 neurons represent the expectation of the presence and absence of a reward. **a** Schematic of reversal learning achieved in one day. **b** Calcium signal in each trial (*top*) and mean calcium traces (*bottom*) for Go and NoGo trials in a session for an example neuron. White ticks indicate licking. O, window opening. R, start of response window. C, window closing. **c** Mean responses of activated and inhibited neurons. Thin gray lines, individual neurons. Thick black lines, mean across the neurons. **d** Proportions of activated, inhibited or non-responsive neurons in O-R period (two-sided Wilcoxon signed-rank test, *p* < 0.05). The proportions were different between Go and NoGo trials (chi-square test, ^***^p = 8.7 × 10^−4^). **e** Heat maps showing the mean calcium responses in Go trials (*left*). Neurons were sorted according to the time of peak response for activated neurons and time of trough for inhibited neurons. Responses of the same neurons in NoGo trials (*right*). **f** Neurons sorted according to responses in NoGo trials (*left*). Responses of the same neurons in Go trials (*right*). **g** Calculation of *d’*. Left: calcium responses in Go (*red*) and NoGo (*blue*) trials, respectively. Right: distribution of calcium responses in Go and NoGo trials. *μ*_*1*_ and *μ*_*2*_, mean calcium responses. *σ*_*1*_ and *σ*_*2*_, standard deviations of the distribution of calcium responses. **h** Distribution of *d’* and significant *d’* values (colored bars, shuffling test, two-sided, *p* < 0.05). **i** Reward- (*red*) and no-reward-selective (*blue*) neurons. Circles, individual neurons. Black circles, the neurons changed their preference. **j** Proportions of reward- (*red*) and no-reward-selective (*blue*) neurons are higher than chance (Chi-square test, *p* = 1.7 × 10^−49^, post-hoc test on adjusted residuals with Bonferroni correction, *p* = 3.9 × 10^−33^ and 4.5 × 10^−21^ for red and blue bar). The broken lines indicate the chance to be Go- or NoGo-preferring on day 2. Proportions of mouse-selective neurons are no greater than chance (*black*). **k** Temporal changes in pointwise *d’* values. Black color indicates values beyond the range of colorbar. Source data are provided as a Source Data file.

To minimize the effect of time differences between sessions, we focused on the session pairs that were conducted 1 day apart, and in which the reward contingency was reversed (Fig. 3a) and behavioral performance was high. We analyzed neurons that were identified on both sessions (1617 neurons from five mice; Supplementary Fig. 4). For each neuron, responses in the high-performance periods (259 ± 36 trials in the high-performance periods) were analyzed (Fig. 3b). In the first session (day 1) of the reversed pair, 34.1% of the neurons were activated and 35.9% were inhibited in response to the reward-associated mouse (Go trials), whereas 27.9% were activated and 41.4% were inhibited in response to the no-reward-associated mouse (NoGo trials; Fig. 3b–d). The proportions were different between the Go and NoGo trials (Fig. 3d). Notably, a large proportion of the activated and inhibited neurons in each trial type (Go or NoGo trials) maintained their responses in the other trial type, as indicated by the broadly similar activity patterns in the heat maps (Fig. 3e, f). Overall, about 70% of neurons maintained the same response in the Go and NoGo trials. A smaller proportion of neurons (~30%) showed different responses in the Go vs. NoGo trials (Supplementary Fig. 6a). To refer to the various responses of the neurons in the Go and NoGo trials, we attributed a response score to each neuron.

Next, we investigated whether the neuronal activity discriminated between the Go and NoGo trials. The difference between the response amplitudes of each neuron in the Go and NoGo trials was evaluated by the discriminability index (*d*′) (Fig. 3g; see Methods). The distribution of *d*′ and the fraction of significant *d*′ on each day were similar before and after reversal learning. On the day before reversal learning (day 1), 21.3% and 12.3% of neurons showed a significant preference for the Go and NoGo trials, respectively (19.7% and 12.0% on day 2, respectively; Fig. 3h). In addition, we examined how the response score of neurons affected their selectivity. On the first day, neurons with different response scores had a different propensity to exhibit Go- or NoGo-preference (Supplementary Fig. 6b). As expected, the neurons with a score of 1, which were activated in Go but not in NoGo trials, had the highest probability (~50%) to be Go-preferring. Interestingly, about 30% of the neurons with a response score of 2 were Go-preferring, indicating that these neurons were activated both in Go and NoGo trials and discriminated the trial types by modulating their response amplitudes. Similarly, the neurons with a response score of 1 or 2 were more likely to be NoGo-preferring than those with a lower response score (Supplementary Fig. 6c).

Because a significant *d*′ value on a single session cannot be ascribed to a stimulus mouse or the presence or absence of the reward, we investigated changes in *d*′ value of each neuron after reversing the reward contingency (Fig. 3i). We found that 42.3% of the Go-preferring neurons on day 1 maintained their preference after the reversal (day 2). Moreover, 32.2% of the NoGo-preferring neurons on day 1 maintained the preference after the reversal (Fig. 3j). As these neurons maintained their Go- or NoGo-preference regardless of the identity of the stimulus mouse, we concluded that these neurons were reward- or no-reward-selective neurons. We also observed that some neurons reversed their Go- or NoGo-trial preference after the reversal learning, and thus maintained their preference for a stimulus mouse across the reversal. However, the proportion of such neurons was not greater than the chance level (Fig. 3j). The examination of the temporal changes in *d*′ values of the reward- and no-reward-selective neurons revealed that the *d*′ values rapidly increased after the window opening, but before a reward was delivered (Fig. 3k). Therefore, these neurons likely represent the expectation of the presence or absence of a reward.

When the same analyses were performed for individual subject mice, reward-specific neurons were found in all five mice. However, no-reward-specific neurons were evident only in the two mice (Supplementary Fig. 7).

Taken together, these results show that dorsal CA1 hippocampal neurons represent the expectation of a reward independent of the associated stimuli. However, we failed to identify neurons representing social identity stably across reversal learning.

### Neural representation of reward expectation is stable over days

The identification of the neurons representing reward expectation is only possible when the neural representations remain stable during reversal learning. Therefore, the reward expectation neurons identified above must have been stable at least for 2 days. We further examined whether these neural representations could be maintained for a longer period.

**Fig. 4:**
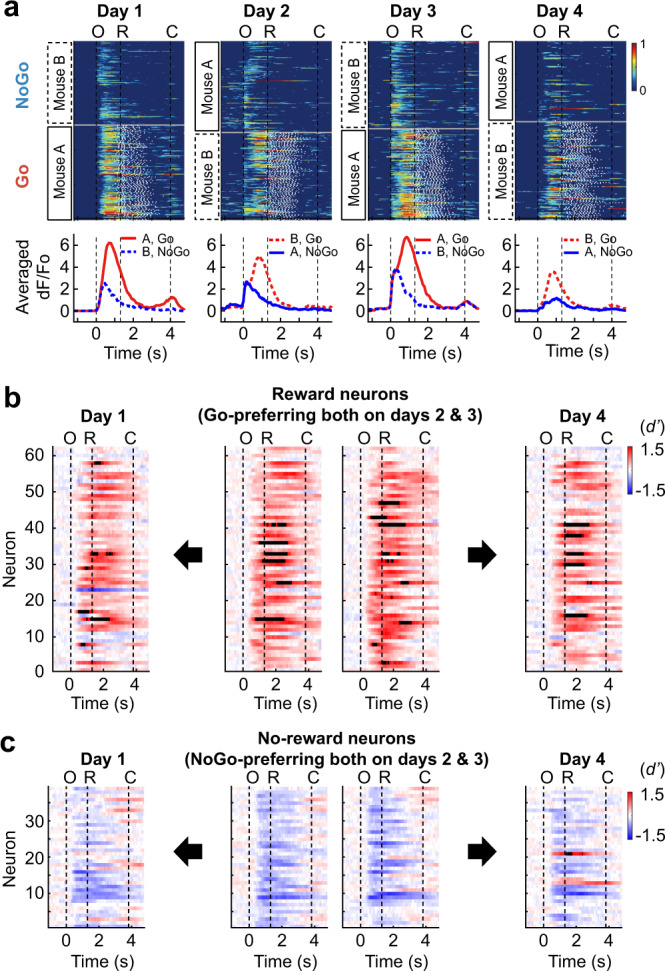
Neural representation of reward expectancy is stable over days. **a** Normalized calcium responses in each trial (*top*) and mean calcium responses (*bottom*) of an example neuron that maintained Go-preference during four consecutive sessions in which reward contingency was reversed daily (811 neurons from three mice; see Supplementary Fig. 4 for the sessions included). Red and blue colors for Go and NoGo trials, respectively. Solid and dashed lines for stimulus mouse A and B, respectively. O, window opening. R, start of response window. C, window closing. **b** Temporal changes in point-wise *d’* value of the reward-selective neurons identified from the pair of sessions on days 2 and 3 (*middle*). Point-wise *d’* values of the reward-selective neurons on days 1 (*left*) and 4 (*right*). **c** Temporal changes in point-wise *d’* value of the no-reward-selective neurons identified from the pair of sessions on days 2 and 3 (*middle*). Point-wise *d’* values of the no-reward-selective neurons on days 1 (*left*) and 4 (*right*). Source data are provided as a Source Data file.

First, we focused on the four consecutive daily sessions during which reward contingency was repeatedly reversed daily, and the behavioral performance was maintained at a high level. In three mice, 811 neurons were tracked over four consecutive days (293 ± 33 trials per session; Fig. 4a). From the pair of sessions on days 2 and 3, we found 63 reward-specific and 37 no-reward-specific neurons. When we checked the Go or NoGo preference of these neurons on day 1, 64.5% of the reward neurons were Go-preferring, and 59.0% of the no-reward neurons were NoGo-preferring (chi-square test, *p* = 1.3 × 10^−32^, post-hoc test on adjusted residuals with Bonferroni correction, *p* < 0.05, Fig. 4b, c). Similarly, 61.3% of the reward neurons were also Go-preferring, and 53.8% of the no-reward neurons were NoGo-preferring on day 4 (Chi-square test, *p* = 3.2 × 10^−32^, post-hoc test on adjusted residuals with Bonferroni correction, *p* < 0.05, Fig. 4b, c). These results demonstrated that the neurons representing the expectation of the presence or absence of a reward displayed a high level of stability over four days.

In addition, we investigated whether the reward- and no-reward-specific neurons could also be identified from the pairs of reversed sessions that were up to 10 days apart and had a high behavioral performance (all the reversed session pairs marked in Supplementary Fig. 4). The proportion of neurons that maintained Go preference across reversal was above the chance level in a majority of the session pairs, although it tended to decline as the time interval increased (Supplementary Fig. 8). However, only one mouse had the neurons that maintained the NoGo preference above the chance level regardless of the time interval between the reversed sessions (Supplementary Fig. 8). Altogether, these results suggest that the expectation of a reward is stably represented by hippocampal CA1 neurons over days.

### Reward expectation neurons are not present in the dorsal CA1 during non-social odor discrimination

**Fig. 5:**
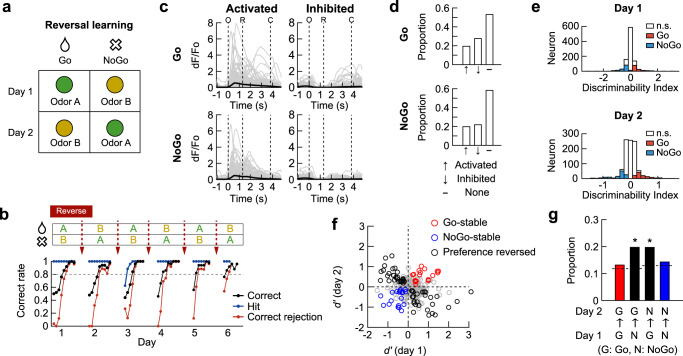
Reward expectation neurons are not present in CA1 during non-social odor discrimination. **a** Schematic of stimulus-reward contingency for a reversed session pair**. b** Learning curves of an example mouse that went through repeated reversal learning. Each filled circle represents the mean performance for fifty trials. **c** Mean responses of activated and inhibited neurons. Thin gray lines for individual neurons. Thick black lines for mean across the neurons. **d** Proportions of activated, inhibited or non-responsive neurons in O-R period (two-sided Wilcoxon signed-rank test, *p* < 0.05). **e** Distribution of *d’* and significant *d’* values (colored bars, shuffling test, two-sided, *p* < 0.05). The neuron with a significant *d’* value was considere*d* as a Go- or NoGo-preferring neuron. **f** Reward- (red) and no-reward-selective (blue) neurons that maintained Go- and NoGo-preference, respectively. Circles indicate individual neurons. Black circles indicate the neurons changed their preference. **g** The proportions of reward- (red) and no-reward-selective (blue) neurons as well as odor-selective neurons are no greater than chance (chi-square test, *p* = 3.4 × 10^−4^, post-hoc test on adjusted residuals, *p* = 6.2 × 10^−4^ for N → G, *p* = 5.3 × 10^−3^ for G → N). The broken lines indicate the chance to be Go- or NoGo-preferring on day 2. The chance levels equal to the proportions of Go- and NoGo-preferring neurons on day 2 in **e**. Source data are provided as a Source Data file.

We explored whether the hippocampal representation of reward expectation is specific to the social memory tasks or is also present during non-social odor discrimination. We trained mice on a non-social odor discrimination task that was identical to the two-mouse discrimination task except for the use of monomolecular odorants as stimuli (Fig. 5a). The mice were repeatedly subjected to reversal learning until they learned the reversed rule in a day (Fig. 5b). We analyzed the activity of the neurons monitored for both days in the reversed session pair that were one day apart, and in which behavioral performance was high.

In the first session (day 1) of the reversed pair, 19.4% of the neurons were activated, and 27.6% were inhibited in response to the reward-associated odor (Go trials), whereas 19.8% were activated and 21.9% were inhibited in response to the no-reward-associated odor (NoGo trials; Fig. 5c, d). The proportion of non-responsive neurons during the non-social odor task was significantly higher compared to the social discrimination task (chi-square test, *p* = 2.9 × 10^−28^ for Go trials, *p* = 5.4 × 10^−23^ for NoGo trials; post-hoc test on adjusted residuals with Bonferroni correction, *p* < 0.05).

Next, we examined whether the neuronal activity discriminated between the Go and NoGo trials. The distribution of *d’* and the fraction of significant *d’* on each day was similar before and after reversal learning. 13.9% and 11.8% of neurons preferentially responded to Go and NoGo trials on day 1 (Fig. 5e). Compared to the social task, the proportion of Go-preferring neurons was significantly lower (chi-square test, *p* = 8.7 × 10^−6^, post-hoc test on adjusted residuals with Bonferroni correction, *p* < 0.05). Furthermore, contrary to the observation in the social discrimination task, the proportion of neurons maintaining their preferential responses to Go or NoGo trials across the reversal in the non-social odor discrimination task was not different from chance (Fig. 5f, g). Instead, a significant fraction of the neurons reversed their Go or NoGo preference and thus maintained their preference for odor stimulus (Fig. 5f, g). These observations suggest that the neurons representing reward expectation appear during social discrimination tasks but not non-social odor discrimination tasks.

We further examined whether information represented at the neuronal population level differed between the social and non-social tasks. SVM decoders were trained with the population activity the day before the reversal and tested whether they could predict Go or NoGo trial types from the population activity the day after the reversal. The SVM decoding accuracy curves deflected upward in the social task indicating the population activity predicts trial types possibly due to the reward expectation signal in dorsal CA1 neurons (Supplementary Fig. 9a). However, the SVM decoding accuracy curves in the non-social task deflected downward suggesting that the population activity patterns better predicted odor stimuli than reward expectation (Supplementary Fig. 9b). The mean deviation of the SVM decoding accuracy for Go or NoGo trial types from chance level was significantly higher for the social than the non-social task (Supplementary Fig. 9c).

### Development of the four-mouse discrimination task

Using the reversal learning approach, we were not able to identify neuronal activity encoding social identities. This result suggests that social identity information might not be available in the dorsal CA1. Alternatively, the neuronal activity representing social identity might have reorganized during the reversal learning, and thus might not be maintained across the reversal. One possible approach to the unambiguous identification of individual mouse-specific neural activity without reversal learning is to assign two stimulus mice for both the reward and no-reward categories. Then, the subject could display the same behavior in response to either stimulus mouse in the same category. Therefore, neuronal responses distinguishing between two reward-associated mice or between two no-reward-associated mice could be ascribed to idiosyncratic differences between them.

**Fig. 6:**
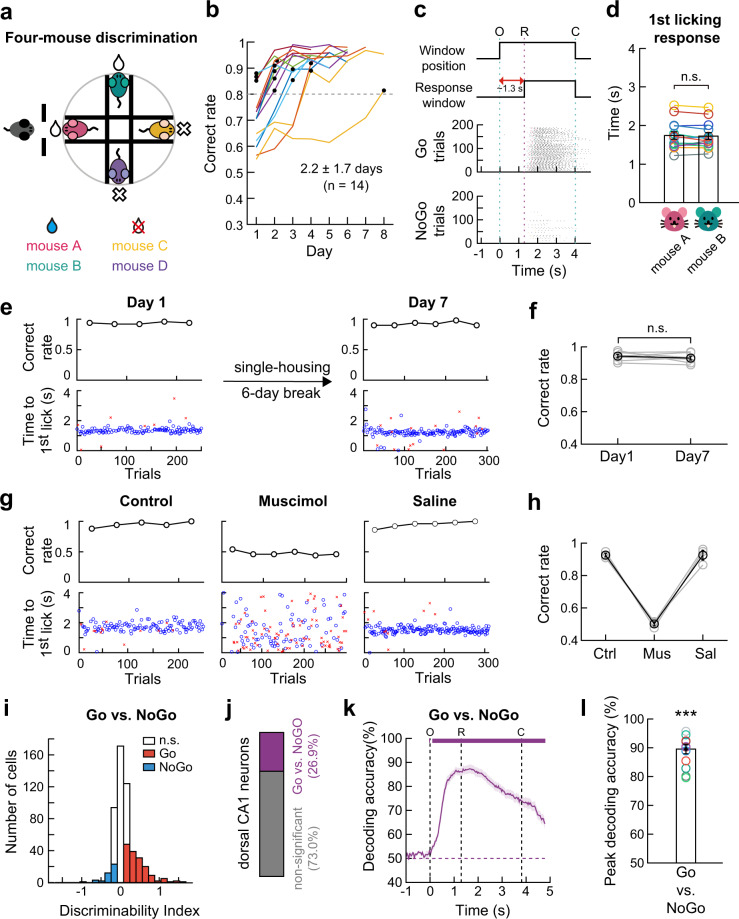
Hippocampal activities in four-mouse discrimination task. **a** Schematic of four-mouse discrimination task. **b** Training time course (*n* = 14 mice). The dashed line indicates the expert criterion. Black circles indicate the first session above the criterion. **c** Task structure (*top*). Raster plots of licking responses of a well-trained mouse in reward. The 1st licking responses to the four stimulus mice were marked with different colors; pink and green for reward-associated mice, yellow and purple for no-reward-associated mice. O, window opening. R, start of response window. C, window closing. **d** The 1st licking responses to each reward-associated mouse were not different (mean ± SEM, two-sided Wilcoxon signed-rank test, *p* = 0.76). Different colors for each subject mouse (*n* = 14). n.s., not significant. **e** Long-lasting memories of individual mice. Task performance (black circles). Blue circles and red crosses for correct and incorrect licks. **f** Group comparison (two-sided Wilcoxon signed-rank test, *p* = 0.54, *n* = 7 mice). Error bars, SEM across mice. **g** Muscimol, but not saline injection decreased the behavioral performance and dispersed the response time. **h** Group comparison of task performance. Ctrl, no injection; Mus, muscimol; Sal, saline. No statistical test made (*n* = 4 mice). Error bars, SEM across mice. **i** Histogram of *d’* calculated within the O-R period for each neuron imaged in an expert mouse. Neurons showing significant *d’* values (shuffling test, two-sided, *p* < 0.05) are color-coded (*red*, Go-selective; *blue*, NoGo-selective). **j** Proportion of neurons discriminating between Go and NoGo trials (*n* = 13 mice). **k** Hippocampal population activity accurately predicted trial types (Go versus NoGo) during the four-mouse discrimination task. The purple trace shows mean (±SEM, shaded area) decoding accuracy across 13 mice. Upper bars indicate the period of significant decoding (Cluster-based permutation test, *p* < 0.05). O, window opening. R, start of response window. C, window closing. **l** The peak decoding accuracy was higher than chance (mean ± SEM, two-sided Wilcoxon signed-rank test, *p* = 4.2 × 10^−6^). Circles for each mouse. Source data are provided as a Source Data file.

Thus, we modified the individual discrimination task into a four-mouse discrimination task (Fig. 6a). In this behavioral paradigm, four equally familiar male littermates were used as stimuli. Therefore, discrimination among them should be based on their individually distinct characteristics. In each trial, one of the four stimulus mice was presented in a pseudo-random order through the interaction window (as described in Fig. 1a and Supplementary Fig. 1). Upon presentation of a reward-associated mouse (mouse A or B), but not a no-reward-associated mouse (mouse C or D), licking the lickport triggered a drop of water reward. After rule learning (discrimination between a stimulus mouse and an empty device), the subject mice proceeded to the four-mouse discrimination task and reached a > 80% correct rate in approximately two days (2.2 ± 1.7 days, 14 mice) (Fig. 6b). In expert mice, the licking responses to the two reward-associated mice were not different; i.e., there was no significant difference in the behavioral response to two mice in the reward or no-reward categories (Fig. 6c, d).

Next, we investigated the duration of social memory by comparing the task performance of well-trained mice before and after a 6-day break. We observed a high correct rate from the beginning of the session after the break, thus confirming the presence of long-lasting memories of the stimulus mice in singly housed mice (Fig. 6e, f).

To verify whether the four-mouse discrimination task also requires hippocampal activity, we reversibly suppressed it by bilaterally injecting muscimol into the dorsal CA1 area. Consistent with the results of the two-mouse discrimination task (Fig. 2a–d), a significant performance impairment was observed in the muscimol group compared with the control and saline groups. (Fig. 6g, h).

We then monitored the activity of hippocampal CA1 neurons during the four-mouse discrimination task using two-photon calcium imaging (336 ± 11 trials per session and 4.6 ± 0.4 sessions per mouse; 13 mice). Similar to the two-mouse discrimination task, 26.9% ± 0.01% of neurons discriminated between reward (Go) and no-reward (NoGo) trials, which was indicated by significant *d*′ values (Fig. 6i, j). Next, we trained an SVM decoder to classify reward and no-reward trials and calculated the decoding accuracy using a leave-one-out cross-validation procedure. SVM decoding analysis in expert mice also showed that hippocampal population activity accurately discriminated between reward and no-reward trials (Fig. 6k, l). These results corroborated the results of the two-mouse discrimination task, in which neural activity in the dorsal CA1 hippocampus provided essential information for performing the task.

### Activity of dorsal CA1 neurons discriminates individual mice

**Fig. 7:**
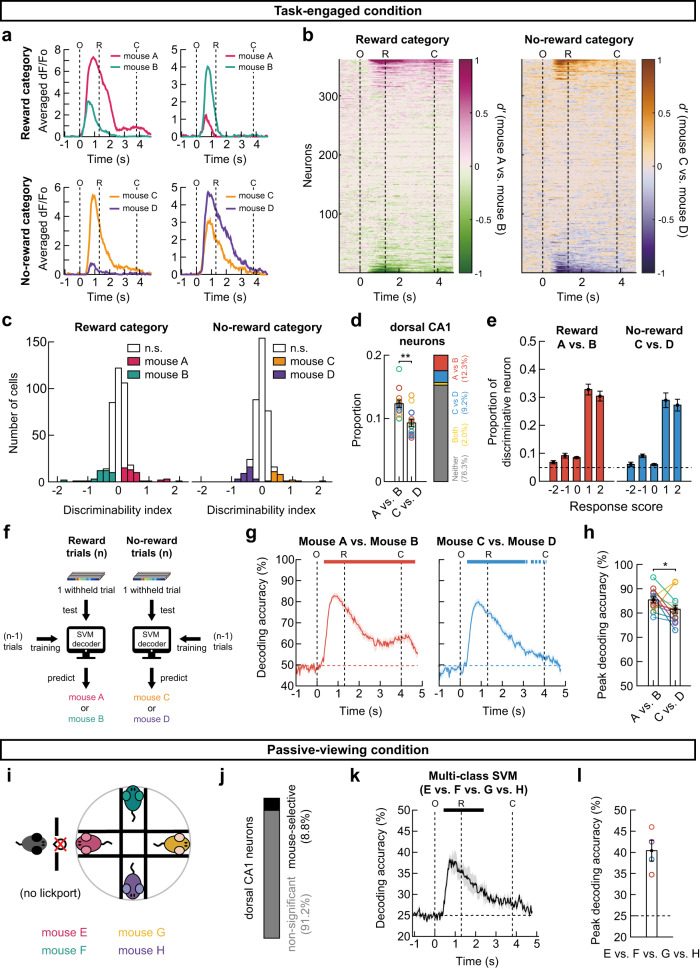
Activity of dorsal CA1 neurons discriminates individual mice. **a** Mean calcium responses of example neurons preferentially responding to each individual stimulus mice. Pink and green colors for reward-associated mouse A and B trials. Yellow and purple colors for no-reward-associated mouse C and D. O, window opening. R, start of response window. C, window closing. **b** Temporal profiles of *d’* for all neurons in an example subject mouse. **c** Distribution of *d’* for the reward-associated (*left*) and no-reward-associated mice (*right*), calculated within the O-C period for all neurons in an example subject mouse. Neurons with a significant *d’* value are color-coded (shuffling test, two-sided, *p* < 0.05). **d** The proportion of mouse identity-selective neurons was higher in reward than no-reward category (mean ± SEM, two-sided Wilcoxon signed-rank test, *p* = 0.0018, *n* = 13 mice). **e** Proportion of mouse identity-selective neurons among the neurons with each response score in reward (*left*) and no-reward (*right*) category (mean ± SEM, *n* = 13 mice). Dashed lines indicate the chance level. **f** Application of support vector machine (SVM) to decode mouse identity-selective information. **g** SVM decoding accuracies of the identity of stimulus mice in the reward (*left*) and no-reward category (*right*). The data are presented as mean ± SEM (shaded area) across 13 expert mice. The upper bars represent the period of significant decoding (Cluster-based permutation test, two-sided, *p* < 0.05). **h** The peak decoding accuracies of individual mice were higher in reward than no-reward categories (mean ± SEM, two-sided Wilcoxon signed-rank test, *p* = 0.046, *n* = 13 mice). **i** Schematic of passive presentation experiments where a novel pair of four conspecifics was presented. **j** Mean proportion of neurons with a significant *d’* (shuffling test with Bonferroni correction, two-sided*, p* < 0.05) obtained from each subject mouse (3–4 sessions) and averaged across four mice. **k** Multiclass SVM decoding accuracies of the stimulus mice in the passive condition (mean ± SEM (shaded area), *n* = 4 mice). **l** Peak decoding accuracies (mean ± SEM). No statistical test made (*n* = 4 mice). Source data are provided as a Source Data file.

Next, we analyzed the hippocampal neuronal responses to individual stimulus mice during the four-mouse discrimination task. We observed that several neurons exhibited different responses to two stimulus mice in the reward as well as in the no-reward categories (Fig. 7a). To quantitatively analyze each neuron’s ability to discriminate between individual mice, we examined the temporal changes in *d*′ values for two stimulus mice either in the reward or no-reward categories (Fig. 7b). Although the majority of CA1 neurons did not discriminate between the stimulus mice, a subset of neurons increased their preference for a stimulus mouse in the same category at ~0.3 s after window opening. Such individual mouse-specific neurons were found both for the reward and no-reward categories. During the stimulus period (O-C), 21.4% ± 0.8% of neurons discriminated between individual mice (Fig. 7c, d); specifically, 12.3% ± 0.6% of neurons discriminated between the reward-associated mice (mouse A vs. mouse B), 9.3% ± 0.6% of neurons discriminated between the no-reward-associated mice (mouse C vs. mouse D), and 2.1% ± 0.3% of neurons discriminated between the individual mice in both categories. The proportion of neurons exhibiting the mouse identity-selective response was significantly higher when the stimulus mice were associated with a reward than when they were not (Fig. 7d; see Supplementary Fig. 10 for each subject mouse). When calculating the proportion of discriminative neurons among the neurons with different response scores, we found that dorsal CA1 neurons with a positive response score (1 or 2) are more likely to be selective for an individual stimulus mouse in both categories (Fig. 7e). Specifically, 30.4% ± 1.7% and 27% ± 2.0% of the neurons with a response score of 1 or 2 were selective for a stimulus mouse within the reward and no-reward categories, respectively. This suggests that about half of the mouse identity-selective neurons responded to both stimulus mice and discriminated between them by modulating their response amplitudes.

We further examined the encoding of individual mouse-specific information at the neuronal population level (Fig. 7f). First, on the reward trials (mouse A and mouse B trials), we performed an SVM decoding analysis using the population of mouse identity-selective neurons. Separate linear decoders were trained to classify the activity pattern in reward trials into either one reward-associated mouse or the other at each time point. The decoding accuracy significantly increased as soon as the subjects encountered the stimulus mouse (Fig. 7g, left). Similarly, an SVM analysis of no-reward trials (mouse C and mouse D trials) showed that the hippocampal neuronal population activity accurately distinguished the two no-reward-associated mice (Fig. 7g, *right*; see Supplementary Fig. 10 for each subject mouse). Moreover, the peak value of SVM decoding accuracy was significantly higher when the stimulus mice were associated with a reward (85.4% ± 1.2%) rather than with the lack of a reward (81.6% ± 1.7%) (Fig. 7h).

Does individual mouse-specific activity of dorsal CA1 neurons appear only during the task? To address this question, we measured the hippocampal activity while a new group of stimulus mice who had not been associated with a reward (mouse E, F, G, or H) was exposed to the subject mice that have previously performed the four-mouse discrimination task (Fig. 7i, *n* = 4). Even without reward association, 8.8% ± 0.8% of dorsal CA1 neurons (a lower proportion compared with the task-engaged condition, but higher than the chance level) could distinguish between the stimulus mice (Fig. 7j). By applying a multiclass SVM analysis (see Methods), we were able to decode mouse identities (mouse E vs. mouse F vs. mouse G vs. mouse H) even in the passive-viewing condition (Fig. 7k, l).

Taken together, these results indicate that dorsal CA1 hippocampal activity accurately discriminated between individual conspecific mice, even in the passive-viewing condition, while encoding more accurate information on reward-associated mice at both the single-neuron and population levels.

### Dynamics of the neural encoding of mouse identity over days

**Fig. 8:**
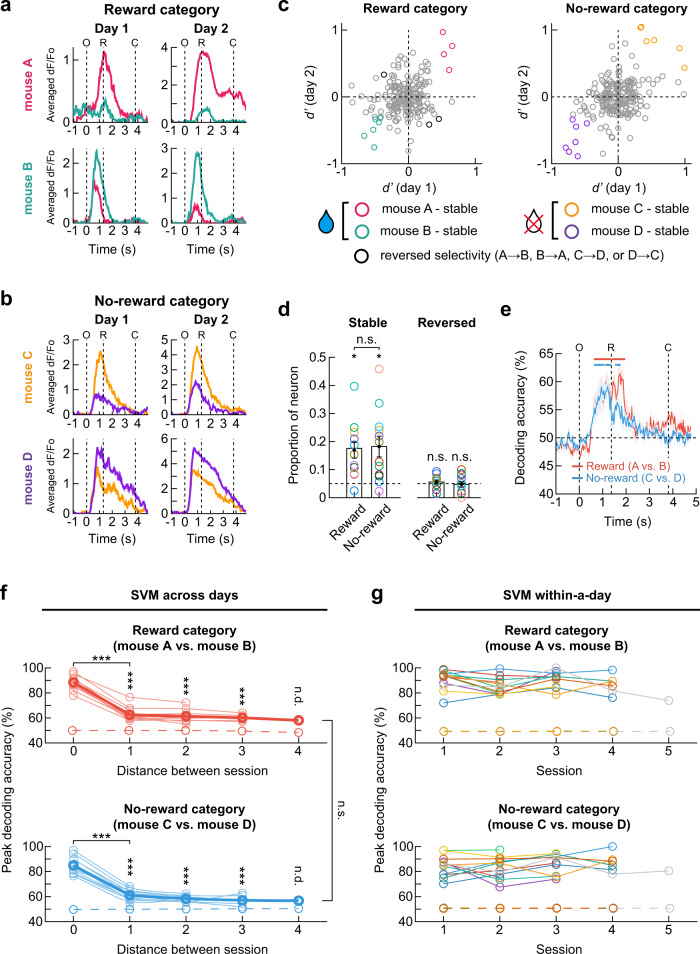
Dynamics of the neural encoding of mouse identity over days. **a**, **b** Example neurons maintaining selective responses to the reward- and no-reward-associated mouse. **c** Changes in *d’* values of each neuron in an example mouse. **d** The proportions of stable (mean ± SEM, two-sided Wilcoxon signed-rank test, *p* = 0.014 and 0.040 for the reward and no-reward category) and reversed neurons (two-sided Wilcoxon signed-rank test, *p* = 0.40 and 0.47 for the reward and no-reward category, *n* = 12 mice). Dashed lines indicate the chance level. No difference between the proportions of stable neurons in the reward and no-reward categories (two-sided Wilcoxon signed-rank test, *p* = 0.89). **e** Neural population activity on day 1 predicted the identity of stimulus mice from population activity on day 2 for an example pair of sessions. Upper bars indicate the period of significant decoding (Cluster-based permutation test, two-sided, *p* < 0.05). **f** Decoding accuracies of individual mice decreased as the time interval between sessions increased. The SVM decoders trained with neuronal activity patterns on day 1 were tested on each trial of day *n* (2 to 5). For the zero distance, within-day decoding accuracy on day 1 was calculated. Across-day decoding accuracies were maintained higher than chance (two-sided Wilcoxon signed-rank test; reward category: *p* = 3.8 × 10^−6^, 1.6 × 10^−5^, 5.1 × 10^−4^ for distance 1, 2, 3, *p* = 3.7 × 10^−5^ for 0 vs. 1; no-reward category: *p* = 6.0 × 10^−7^, 6.4 × 10^−5^, 9.1 × 10^−4^ for distance 1, 2, 3, *p* = 2.2 × 10^−9^ for 0 vs. 1). Across-day decoding accuracies were similar between reward and no-reward category (two-sided Wilcoxon signed-rank test, *p* = 0.71, *n* = 12 mice). Thin lines for each mouse. Thick lines for mean. **g** Within-session decoding accuracies of the identity of stimulus mice were stably high (*n* = 12 mice). Dashed lines in **f** and **g** represent decoding accuracies obtained from shuffled data. O, window opening. R, start of response window. C, window closing. ^*^p < 0.05, ^**^p < 0.01*,* ^**^^*^p < 0.001, n.s., not significant. n.d., not determined. Source data are provided as a Source Data file.

Lastly, we sought to determine the stability of the hippocampal activity that discriminated between individual mice. To address this question, we tracked the activity of the same neurons over consecutive daily sessions (54 pairs from 12 mice; one mouse was excluded because it was not repeatedly imaged for successive days). Interestingly, we observed that several neurons maintained their preferential response to one stimulus mouse over the other (either mouse A vs. mouse B or mouse C vs. mouse D) for two consecutive days (Fig. 8a, b). Accordingly, to evaluate the stability of individual discriminability, we compared each neuron’s *d*′ values on the first and second days. Although a large proportion of dorsal CA1 neurons changed the value and sign of *d*′ over two consecutive days, some neurons maintained the significance of *d*′ value and its sign, indicating that these neurons preserved their preference toward one mouse over the other (Fig. 8c). This result suggests that these neurons preserved their preference to one mouse over the other during two consecutive days. Although the fraction of stable neurons was significantly higher than the chance level in both the reward and no-reward categories, the fractions of the stable neurons in the reward (16.9% ± 2.8%) and no-reward (18.2% ± 3.7%) categories were similar (Fig. 8d). However, the fraction of neurons that changed their mouse selectivity over two days (i.e., mouse A(C) to B(D) or vice versa) did not exceed the fraction expected by chance (5.6% ± 0.7% for the reward category and 4.7% ± 1.0% for the no-reward category; Fig. 8d).

Next, we conducted an SVM decoding analysis on the session pairs to assess the stability of the individual mouse-specific information at the neuronal population level. For both the reward and no-reward categories, the SVM decoder trained with the neuronal activity pattern on the first day could successfully decode mouse identity from the population activity on the other day up to four days apart (Fig. 8e, f). As expected, the decoding accuracy increased upon presentation of the stimulus mouse and decreased during reward consumption (Fig. 8e). However, the peak value of the across-day decoding accuracy was significantly lower compared with the within-day decoding accuracy (Fig. 8f). The across-day decoding accuracies of the reward and no-reward category were similar. Considering the fact that the within-day SVM decoding accuracies for individual mice were high (Fig. 8g), we inferred that an ever-changing subset of dorsal CA1 neurons, which allow a certain degree of overlap in mouse-selective neurons, contribute to the stable encoding of individual-specific information over days. Similarly, across-day decoding accuracy for trial types (Go or NoGo) decreased as the interval between sessions increased, while within-day decoding accuracy maintained high (Supplementary Fig. 11a, b).

Taken together, our results suggest that hippocampal CA1 neurons dynamically represent social identity with some level of long-term stability at both the single-cell and population levels, which provides a critical insight into cognitive flexibility to process social information in the dorsal CA1 hippocampus.

### Activity of dorsal CA1 neurons discriminates non-social odors

**Fig. 9:**
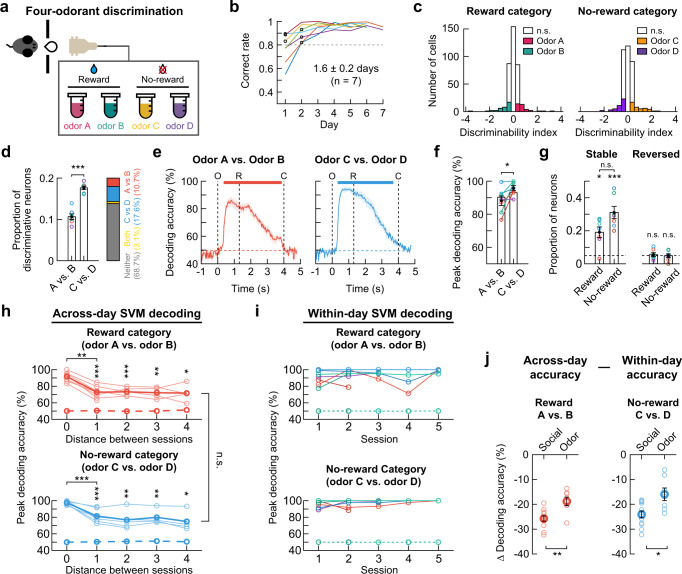
Activity of dorsal CA1 neurons discriminates non-social odors. **a** Task schematic. **b** Training time courses. Black circles, the first session above the criterion (dashed line). **c** Distribution of *d’* for all neurons in an example mouse. Neurons with a significant *d’* are color-coded. **d** Proportion of odor -selective neurons (mean ± SEM, two-sided Wilcoxon signed-rank test, *p* = 5.8 × 10^−4^, *n* = 7 mice). **e** Odor identity decoding (mean ± SEM (shades), *n* = 7 mice). The upper bars, the period of significant decoding (Cluster-based permutation test, two-sided, *p* < 0.05). **f** A higher decoding accuracy in no-reward category (mean ± SEM, two-sided Wilcoxon signed-rank test, *p* = 0.025, *n* = 7 mice). **g** Proportions of stable (two-sided Wilcoxon signed-rank test, *p* = 0.017 and 5.8 × 10^−4^ for the reward and no-reward categories) and reversed neurons (*p* = 0.40 and 0.47 for the reward and no-reward categories, *n* = 7 mice) during two consecutive days. Dashed lines, chance level. Similar proportions of stable neurons in both categories (two-sided Wilcoxon signed-rank test, *p* = 0.053). Bars, mean ± SEM. **h** Lower decoding accuracies of odors with longer session intervals (two-sided Wilcoxon signed-rank test; reward category: *p* = 2.1 × 10^−4^, 5.6 × 10^−3^, 0.008, 0.03 for distance 1, 2, 3, 4, *p* = 0.001 for 0 vs. 1; no-reward category: *p* = 4.1 × 10^−5^, 0.006, 0.008, 0.03 for distance 1, 2, 3, 4, *p* = 7.8 × 10^−4^ for 0 vs. 1). Across-day decoding accuracies were similar between the categories (two-sided Wilcoxon signed-rank test, *p* = 0.71, *n* = 12 mice). Thin lines for each mouse. Thick lines for mean. **i** Within-session decoding accuracies. Each color, different mouse (*n* = 7 mice). Dashed lines (**h** and **i)** for shuffled data. **j** The within-day vs. across-day decoding accuracy difference (distance 0 vs. 1) was greater in the social task (mean ± SEM, two-sided Wilcoxon singed-rank test, *p* = 0.0072 and 0.017 for reward and no-reward category). ^*^p < 0.05, ^**^p < 0.01, ^***^p < 0.001, n.s., not significant. Source data are provided as a Source Data file.

Next, we trained seven mice on a four-odor discrimination task to test whether dorsal CA1 neurons discriminate between non-social odors in the reward or no-reward category (Fig. 9a, b). Similar to the four-mouse discrimination task, 31.4 ± 0.8% of neurons discriminated between two odors (Fig. 9c, d); specifically, 10.7 ± 0.7% of neurons discriminated between the reward-associated odors, 17.6 ± 0.4% of neurons discriminated between the no-reward-associated odors, and 3.1 ± 0.5% of neurons discriminated between the odors in both categories. The proportion of odor-selective neurons was higher when the stimulus was associated with no-reward than reward (Fig. 9d). An SVM decoding analysis showed that hippocampal neuronal population activity accurately discriminated between two odors in the reward and no-reward categories (Fig. 9e). The peak value of SVM decoding accuracy was higher in the no-reward category (Fig. 9f).

Lastly, we checked the stability of the odor-specific hippocampal activity by monitoring the same neurons over consecutive daily sessions. The proportion of neurons that maintained their preference for one odor over the other was significantly higher than the chance in both the reward (19.1% ± 3.2%) and no-reward (31.1% ± 3.7%) categories (Fig. 9g). However, the proportion of neurons that changed their odor selectivity (i.e., odor A(C) to B(D) or vice versa) was not different from the chance level (5.5% ± 1.2% for the reward category and 4.9% ± 1.2% for the no-reward category; Fig. 9g). Next, we conducted an SVM decoding analysis to assess the stability of the odor-specific information at the neuronal population level over five consecutive days. For both the reward and no-reward categories, the SVM decoder trained with the neuronal activity pattern on the first day could successfully decode odor identity from the population activity on the other day up to 4 days apart (Fig. 9h). The across-day decoding accuracy was significantly lower than the within-day decoding accuracy (Fig. 9h), while the within-day SVM decoding accuracies for odors maintained high (Fig. 9i). The difference between the within-day and across-day decoding accuracies was more prominent for the social than non-social odor discrimination (Fig. 9j). In addition, across-day decoding accuracy for trial types (Go or NoGo) decreased as the interval between sessions increased, while within-day decoding accuracy maintained high (Supplementary Fig. 11c, d).

Altogether, these results suggest that dynamically changing populations of dorsal CA1 neurons represent both social and non-social odor stimuli with faster changes in social representations.

## Discussion

Although individual recognition is achieved via the investigation of individually unique characteristics such as the face, vocalization, or odor in different species^43–45^, rodents recognize individuals based on their unique olfactory signatures^46–48^. During the social investigation, rodents display vigorous sniffing toward the face, flank, and anogenital areas of their social counterparts^49,50^. However, it is unclear whether social recognition requires vigorous sniffing of various body parts. Our results indicate that a brief (approximately 0.5 s) olfactory investigation of the nose of the social counterpart is sufficient. Therefore, individual recognition seems to occur at the beginning of the investigatory sniffing, and further sniffing toward different body parts may continue to collect other individual-specific information or play other functions. Notably, a previous study demonstrated that sniffing not only collects olfactory information, but also delivers social hierarchy information^50^.

In social novelty preference paradigms, social memory lasts no more than 24 h in single-housed rodents^6,33^. In contrast with those results, we observed that the memories of individual mice were maintained in single-housed mice for at least 6 days. This discrepancy could be attributed to the appetitive conditioning used in our behavioral paradigm; water restriction may have increased the need to maintain social memories for a longer period. Another possibility is that our paradigm and the social novelty preference paradigms may test different types of recognition memory retrieval, i.e., conscious recollection and a sense of familiarity, respectively^39^. The decreased social investigation toward a familiar conspecific in novelty preference paradigms may rely on a sense of familiarity, whereas our paradigm requires mice to consciously recollect individual-specific information regarding reward association, thus leading to different strengths of social memories. Lastly, the lack of social memory in the social novelty preference test can be interpreted differently. Single-housed animals may feel lonely or bored due to the deprivation of social interaction. Thus, their increased desire for social interaction may encourage them to investigate familiar conspecifics as much as novel ones even though they have intact memories of the familiar conspecifics.

In the present study, we demonstrated that both the presence and absence of a reward are represented by dorsal CA1 neurons. While reward-selective neurons were present in all mice tested, the presence of no-reward neurons was evident only in two mice that had learned the reversed rule multiple times. Thus, whether no-reward neurons appear only when the hippocampus adapts to flexible changes in reward contingency is of interest. This needs to be confirmed by a larger cohort of animals. We identified these neurons based on their activities before the water reward was delivered. Therefore, we speculate that these neurons most likely code for predicting the presence and absence of a reward, rather than for receiving a reward or not. These findings expanded the recent observation that dorsal CA1 and the subiculum contain a dedicated population of reward-predictive neurons^20^. In the previous study, reward neurons were found in mice navigating virtual environments, whereas such reward-predictive neurons were present in head-fixed mice engaged in a non-spatial task in our study. It should be noted that there is an alternative explanation for reward-selective neurons. As soon as recognizing the stimulus mouse, subject mice also anticipate whether they will lick or not after a grace period. Therefore, reward-selective neurons can be interpreted as responding to the anticipation of a licking behavior rather than a reward.

It is often challenging to dissociate such reward-related neural responses from those evoked by concurrent sensory stimuli. Using a reversal learning approach, we could unambiguously identify reward- and no-reward-specific neurons that maintained their selectivity regardless of the associated stimuli. Because learning often reorganizes neural representation of the task context^51–53^, the identification of reward-related neurons during reversal learning indicates that these neurons are highly stable. In fact, the identified reward- and no-reward-specific neurons exhibited a high probability of maintaining their specificity throughout repeated reversal learning. Furthermore, reward-selective neurons could be identified in the pairs of reversed sessions that were several days apart. These observations suggest that reward-related information in the dorsal CA1 is represented by highly stable subsets of neurons across different contexts^20^.

The stability of the reward- and no-reward neurons is in sharp contrast with the rapid changes in the neuronal population representing individual stimulus mice. Upon reversal learning, the proportion of neurons that maintained the mouse-specific response was no greater than chance. This suggests that the neural representations of individual mice were completely reorganized at the neuronal level during reversal learning, although we cannot exclude the possibility that individual-specific information may maintain to some extent at the population level. In the four-mouse discrimination task, which does not require reversal learning to identify individual mouse-specific neural activity, we found that dorsal CA1 neurons discriminated between stimulus mice in the reward or no-reward category. In addition, the population activity of the dorsal CA1 was able to predict the identity of stimulus mice with high accuracy within each task session. Considering that the odors from male littermate mice are largely overlapping, dorsal CA1 neurons may contribute to individual recognition by selectively responding to unique odor components or combinations of odors from stimulus mice. Despite the high predictability of social identity in each session, the neural representation of individual mice was substantially altered even on the next day, even though there was no apparent change in the task and behavioral performance. While social representations change more rapidly than non-social ones, such a drift in the neural representation in a similar time frame has been reported in CA1 place cells in the same environment^54,55^. This observation suggests that the stable representations of an individual mouse may be stored in other brain regions, possibly including the ventral CA1, which has been found to be important for social memory formation^8,9^, and the dorsal CA2 and medial prefrontal cortex, where social identity can be decoded^56–58^. These brain areas may provide an ever-changing population of neurons in the dorsal CA1 with individual mouse-specific information.

In the present study, we trained animals to discriminate between reward and no-reward-associated familiar conspecifics. One major caveat of this approach is that discrimination might be made between reinforced and non-reinforced stimuli^59^. We avoided this issue by assigning two stimulus mice for both the reward and no-reward categories and demonstrated that two equally reinforced or non-reinforced stimulus mice in the same category could be discriminated by hippocampal neural activity. Interestingly, although the no-reward-associated stimulus mice were not reinforced by a reward during the training, they were slightly less, but similarly discriminable by CA1 neuronal activity compared with the two reward-associated stimulus mice. These results indicate that the individual discriminability of hippocampal neurons is not simply a consequence of the reinforcement process. In agreement with this speculation, we observed that dorsal CA1 neurons distinguished between conspecifics even in the absence of a task.

We observed that inhibiting dorsal hippocampal activity impaired social, but not non-social odor discrimination task performance. These findings are consistent with the previous reports that the hippocampus is not necessary for non-social odor perception^60–64^. Especially in head-fixed mice performing a Go-NoGo non-social odor discrimination task, suppression of CA1 activity partially impairs behavioral performance during the learning phase, but this effect disappears in well-trained mice^65^. Despite the different involvement of dorsal CA1 neurons in social and non-social tasks, we found that CA1 neurons represented odor stimuli regardless of whether they are social or not. Therefore, it is unclear whether dorsal CA1 neurons are required to perform individual discrimination tasks as they represent social stimuli. Interestingly, reward-selective neurons were found only during social tasks. To perform the task, subject mice must associate a social stimulus with a water reward that is not inherently social. Thus, the role of dorsal CA1 may be to associate a reward with individual conspecific mice, and the presence of reward-selective neurons may indicate the involvement of dorsal CA1 in the retrieval of stimulus-reward associations. It is intriguing why dorsal CA1 neurons acquire reward selectivity when rewards are associated with social stimuli.

Recent studies have shown that the dorsal CA1 is not necessary for discriminating between a novel mouse and a familiar mouse^9,30,31^. Although the current study did not directly test whether the dorsal CA1 is critical for discriminating between a novel and a familiar mouse, our results clearly indicate that the dorsal CA1 is required for discriminating between two familiar mice, consistent with a previous reports^7^. As discussed previously, our task was different from social novelty preference paradigms in several aspects, such as the need to maintain social memory or the strength of the social memory established during the tasks. Thus, the differences in social memory tasks may be responsible for the different hippocampal subregions that were recruited. A previous study also demonstrated that social engram exists in the ventral CA1, and that associating the engram with foot shocks or cocaine injections elicits an aversion to or preference for the mouse with which the engram is labeled. However, such memory inception fails in neurons in the dorsal CA1^9^, suggesting the absence of social memory in this region. We observed that the pattern of individual-specific activity in the dorsal CA1 dynamically evolved between task sessions, although some neurons maintained their mouse selectivity. Therefore, memory engram approaches may not be as effective in the dorsal CA1 hippocampus.

In addition to the differences mentioned above between our individual discrimination paradigm and the social novelty preference paradigms, we speculate that several advantageous features of our behavioral paradigm may also have helped us identify individual mouse-specific activity. First, both the subject and the stimulus mice were head-fixed, thereby removing several confounding factors. For example, the distance between the noses of the interacting mice was identical in each trial, and the axes along their bodies were consistently aligned. In addition, spatial factors were excluded. Second, and more importantly, using two stimulus mice both for the reward and no-reward categories allowed us to identify individual-specific activity independent of the activities related to other factors, such as the prediction of a reward and subsequent behavior. Third, the regular temporal structure of our task unequivocally specified the time window of social investigation during which individual-specific neuronal responses were determined. Lastly, identical social interactions were repeated hundreds of times during a session, which may have increased the statistical power of the analysis.

## Methods

### Animals

Male C57BL/6 J wild-type mice (The Jackson Laboratory; 2–11 months old) were used for subjects in behavioral tests and were also used for stimulus mice. Male GCaMP6f mice (C57BL/6J-Tg (Thy1-GCaMP6f) GP5.17Dkim/J; stock number, 025393; The Jackson Laboratory; 2–10 months old) were prepared for two-photon imaging experiments. Four of them were not proceeded to imaging experiments due to poor image quality or significant motion artifact and were used only for behavioral tests. After surgery, the subject mice were singly housed in individual cages throughout the experiments. Stimulus mice used in each behavioral session were male littermates so that they were the same in sex, age, and genetic makeup. However, the stimulus mice were not littermates of the subject mice. In addition, the level of familiarity of the stimulus mice with the subject mouse was maintained similarly by allowing the subjects and the stimulus mice to interact only during task sessions. Therefore, the stimulus mice used in each task session differed only in their individually unique characteristics. For two-mouse discrimination tasks, two stimulus mice were housed separately in the same cage partitioned with a transparent and multiperforated wall which allowed odors in each partition to be mixed. For four-mouse discrimination tasks, each stimulus mouse was housed individually after head bar surgery. The mice were maintained on a 12-h light/dark cycle at 22 °C with 50% humidity with food *ad libitum*. All procedures used in this work were approved by the Institutional Animal Care and Use Committee of the Institute for Basic Science (Daejeon, South Korea).

### Surgery

The mice were anesthetized with isoflurane (1–2%) and were mounted in a stereotaxic frame (Narishige, Japan) while maintaining body temperature at 37 ˚C using a heating pad (DC temperature control system, FHC Inc., Bowdoin, ME, USA). Ophthalmic ointment was applied to the eyes to prevent drying during surgery. Before scalp incision, 0.5% bupivacaine in saline was injected for local anesthesia. For the mice used for two-photon imaging, a circular craniotomy of 3.2 mm diameter was made centered at 1.8 mm posterior to bregma and 1.4 mm lateral to the midline over the right hemisphere. The parts of the cerebral cortex and the corpus callosum above the dorsal hippocampus were removed by aspiration. Then a cranial window was implanted into the craniotomy. The cranial window was composed of a custom-made stainless-steel cannula (outer diameter of 3.2 mm, inner diameter of 2.8 mm, and height of 1.8 mm) and a 3 mm diameter coverslip (CS-3R-0, Warner Instruments, Hamden, CT, USA) attached at the bottom of the cannula by UV-glue. Four protrusions (height of 0.5 mm) from the top of the cannula touched against the surface of the skull when the cannula was inserted into the craniotomy. Thus, the depth of the cannula inserted below the surface of the skull was 1.3 mm. The cranial window and a stainless-steel head bar were affixed to the skull using light-cured dental composite and dental acrylic.

### Social discrimination apparatus

The social discrimination apparatus was built in a behavior box with the interior lined with sound-proof foam. The box (64 (w) × 64 (l) × 60 (h) cm) had LEDs to illuminate the interior. There was a wall (35 (w) × 14 (h) cm, placed 20 cm above the floor) dividing the inner space into two compartments. The wall was extended to the floor of the box with a flexible polyvinyl chloride curtain to block the odor diffused from the presented mice. On one side of the box, a head-fixing device for a subject mouse was placed. On the other side, there was a circular platform (20- or 24 cm diameter). A stepper motor (SBC-NK245-03AT, Motorbank, South Korea) was used to rotate the platform to an angular position relative to a reference position on the platform. The reference position was sensed by an infrared beam breaker (SEN-00241, SparkFun Electronics, Niwot, CO, USA). There were removable head-fixing devices (two devices at the opposite sites for two-mouse discrimination tasks and four devices 90 degrees apart for four-mouse discrimination tasks) where stimulus mice were mounted facing outward. The head-fixing devices and mounting locations on the platform were randomly assigned to each mouse for each session. The wall had a rectangular window (3 (w) × 2.3 (h) cm) through which the subject mouse faced one of the stimulus mice. The window was opened and closed by a piece of cardboard attached to a servo motor (MEDS15, Makeblock Co. Ltd, Shenzhen, China). When a subject mouse and a stimulus mouse were facing each other through the window, the noses of the two mice were 1 ~ 2 cm apart. A lickport mounted on a manual three-axis micromanipulator was placed in front of the subject mouse. The lickport was made of a stainless-steel tube (1.3 mm outer diameter) and also used as a lick sensor itself^66^. Water rewards were delivered through a tube into the lickport from a water reservoir (a 10 ml syringe) placed 50 cm above the lickport using gravity. A solenoid valve (161T011, NResearch Inc., NJ, USA) was used to control the reward. An Arduino was used to control the stepper motor for the circular platform. Another Arduino was used to control the servo motor and the solenoid valve and record signals from the beam breaker and the lickport. A graphic user interface written with MegunoLink Pro (Hamilton, New Zealand) was used to display task progress and record data. There were two cameras in the behavior box. One was positioned in the middle of the ceiling for the overall view of the experiment. The other one was on the side for monitoring the behavior of subject mice.

### Individual discrimination task

After at least a week of recovery from surgery, subject mice were put on a water-restriction schedule (~1 ml water daily; ~80% of normal body weight). Stimulus mice were also put on a mild water restriction (1.6 ml daily after a task session), which led to infrequent urination during head-fixation, making them sit comfortably during task sessions. After three days of handling and habituation, training began with rule-learning sessions during which the subject mice learned to discriminate a mouse (reward-associated stimulus) from an empty head-fixing device (no-reward-associated stimulus). At the start of a trial, the rotating platform was located at a neutral angular position where both the stimulus mouse and the empty head-fixing device were at 90 degrees from the interaction window. One second after the start, the stepper motor brought one of the two stimuli to the interaction position in front of the window by rotating the circular platform by 90 degrees with an angular speed of 36 deg/s. Then the interaction window opened. The stimulus presented in each trial was pseudo-randomly determined with constraints that the same stimulus was not presented for more than three consecutive trials, and the difference in accumulated numbers of trials for each stimulus was no more than 10. After the window opened, there was a delay period (0.8–1.3 s; 1.3 s for most mice) during which the reward controlling valve remained inactive, thereby encouraging the subject mouse to refrain from licking during this period. A fixed delay was used in all sessions of each mouse. The delay was followed by a response window in which the reward valve was active. The response window lasted for 2.7–3.2 s until the interaction window closed. Therefore, the interaction window remained open for 4 s. The first licking in the response window upon presentation of the reward-associated stimulus triggered the delivery of a drop of water (~4 μl) but not upon the presentation of the no-reward-associated stimulus (Go/NoGo task). We did not punish incorrect NoGo responses (misses) and incorrect Go responses (false alarms). Hit, miss, false alarm, and correct rejection was determined by licking responses in the response window. One second after the window closing, the platform turned 180 degrees to the other neutral position to begin the next trial. By doing so, the direction of rotations of a stimulus to the interaction position in each trial was randomized to prevent the subject mice from performing the task by picking up on a subtle difference in the noise from the stepper motor rotating the platform in different directions. Trials were repeated every 12 s. The subject mice performed approximately 300 trials per session and one session per day. Within a few days, the subject mice learned the rule and responded correctly at a rate of ~80%. Then the subject mice proceeded to individual discrimination sessions. The two-mouse discrimination task was identical to the rule-learning sessions except for using two mice as stimuli. One mouse was assigned as a positive stimulus (reward-associated), and the other as a negative stimulus (no-reward-associated). The stimulus mouse used in the rule learning was not used again for the individual discrimination sessions for the same subject mice. By positioning the lickport as far from the subject mouse as possible, we could discourage impulsive licks upon presenting the negative stimulus^67^. For the four-mouse discrimination task, each stimulus mouse was mounted equally apart along the perimeter of the rotating platform. At the start of a trial, a neutral position between two neighboring stimulus mice was placed at the interaction position. Then the platform rotated 135 degrees, either clockwise or counterclockwise directions, to bring a stimulus mouse to the interaction position. The direction of rotation was randomly chosen. After the end of a trial, the platform rotated 45 degrees in the randomly chosen direction. In this way, the presentation order of the stimulus mice was determined pseudo-randomly. The subject mice performed 400–600 trials per session. Some mice were trained with slightly different training protocols, for example such as training without rule learning. Training using the earlier protocols took a bit longer to reach a correct rate above 80% and was not included in the learning curves in Figs. 1d and 6b.

### Testing long-term memories of individual stimulus mice

To investigate how long the memories of individual mice lasted, we used two approaches in the two-mouse discrimination task. First, with well-trained subject mice, individual discrimination performance was compared before and after a 72 h break during which the subject mice were maintained in the home cage. Second, we reversed the reward contingency after the 72 h break and tested if behavioral performance started below the chance level due to the memories of the individual stimulus mice and the previous reward contingency. Behavioral performance was compared during the 100 trials before and after the 72 h break for the reversal test. In the four-mouse discrimination task, the performance was compared before and after a 6-day break. Note that all subject mice were singly housed during the experiments.

### Individual discrimination in the light and dark conditions

To test whether visual information was necessary for discriminating between the stimulus mice, the behavioral performance of well-trained subject mice (8 mice) in the light condition was compared with that in the dark condition (4 and 0.4 lx, respectively). In six mice, the LEDs in the behavior box were turned on and off every 50 trials. In the other two mice, light and dark conditions were alternated irregularly. When the light/dark comparisons were made in more than one session (2 to 4 sessions in 7 out of 8 mice), mean performance in the light and dark conditions were calculated in each mouse before group comparison.

### Non-social odor discrimination task

The task was conducted in the same social discrimination apparatus with a custom-made odor delivery system. The task structure was identical to the social discrimination tasks except that two odor cues (citral (W230308) and 1-butanol (537993); 1:1000 diluted with mineral oil; Sigma-Aldrich, USA) or four odor cues (citral (W230308), 1-butanol (537993), isoamyl acetate (W205532), L-carvone (W224901); 1:1000 diluted with mineral oil; Sigma-Aldrich, USA) were used as stimuli. The odors were presented through a polyurethane tube (1/8 inch diameter) placed 2 cm apart from the nose of the subject mice.

### Muscimol injection

A day before the start of muscimol or saline injection experiments, small bilateral craniotomies (~1 mm diameter) were made and covered with Kwik-Cast (WPI, Sarasota, FL, USA) under anesthesia with isoflurane (1–2%). On the day of injection experiments, the mice were anesthetized with isoflurane (1–2%) and a glass micropipette (30 μm tip diameter, beveled at 30° by using a microgrinder (EG-44, Narishige, Japan) or cut by using a microforge (MF-900, Narishige, Japan)) was stereotaxically inserted into the dorsal hippocampi (1.8 mm posterior to bregma, ±1.4 mm lateral to the midline, and 1.3 mm deep from the dura). 200 nl of either muscimol (1 mg/ml, dissolved in saline; 0289, Tocris, UK) or saline was bilaterally injected (2 nl/s) through the micropipette using a hydraulic injection system (Nanoject III, Drummond, Broomall, PA, USA). Behavioral tasks were conducted about an hour after the injection. When visualizing the spread of muscimol, BODIPY-conjugated one (muscimol-BODIPY TMR-X, 0.5 mg/ml, dissolved in 400 nl saline; M23400, Thermo-Fisher, USA) was injected before the last task session for each subject mouse. Immediately after the end of individual or non-social odor discrimination tasks, the subject mice were sacrificed by cardiac perfusion under ketamine-xylazine anesthesia (120 mg/kg ketamine; 10 mg/kg xylazine).

### Histology

To confirm the position of cranial window implantation or the drug infusion sites, histological analysis was conducted after the last experiment for each mouse. The subject mice were anesthetized by injecting ketamine-xylazine mixture (120 mg/kg ketamine; 10 mg/kg xylazine), and were sacrificed and fixed by cardiac perfusion with saline and then 4% paraformaldehyde (PFA) (T&I biotechnology, South Korea). Then the brain was kept submerged in 4% PFA overnight at 4 °C. On the next day, coronal hippocampal sections (thickness, 100 μm) were obtained by using a vibratome (VT1200-S, Leica, Germany). After brief washing with 0.1% Triton X-100 dissolved in phosphate-buffered saline (PBST), brain slices were incubated for 5 min with DAPI solution (1:1000 dilution in 0.1% PBST) for staining nuclei and then were mounted. The fluorescence of GCaMP6f or BODIPY-conjugated muscimol was imaged using a confocal laser scanning microscope (Ti-E Eclipse with PFS; Nikon, Japan).

To check the expression of GCaMP6f in hippocampal CA2 subregion, Rgs14-positive neurons were stained in two Thy1-GCaMP6f mice. After the brain was extracted and fixed in 4% PFA overnight, 30-μm sections were prepared. Brain slices were incubated in a blocking solution (5% normal goat serum, 0.1% PBST) for two hours at room temperature. Subsequently, slices were incubated with primary antibody (mouse monoclonal anti-Rgs14 diluted 1:50; 75–170; Neuromabs) in a blocking solution overnight at 4 °C. After the sections were washed three times for 15 min with 0.1% PBST at room temperature, they were incubated with secondary antibody (Alexa Fluor 647-conjugated anti-mouse IgG antibody diluted 1:800; ab150115; Abcam) in the blocking solution for 2 h. Slices were rinsed three times for 15 min with 0.1% PBST, stained with DAPI (1:1000 dilution in 0.1% PBST), and then mounted on microscope slides. The fluorescence imaging was performed with an inverted confocal microscope (Ti-E Eclipse with PFS; Nikon, Japan) using a CFI Plan Apo 20× (NA 0.75) and CFI Plan Fluor 40× (NA 0.75) objective lenses.

### Two-photon imaging

Changes in GCaMP6f fluorescence in the dorsal CA1 hippocampus were imaged using a two-photon microscope (Vivoscope, Scientifica, UK). Fields of view (800 μm x 800 μm, 512 × 512 pixels) were scanned at 30 frames/s with an excitation light of 925 or 940 nm from a femtosecond pulsed laser (Vision II, Coherent, UK). Emission lights were collected through a 16X water immersion lens (0.8 NA, 3.0 mm WD, CFI75 LWD, Nikon, Japan) mounted at 5° on a tiltable nosepiece. To prevent ambient light from entering into the emission path through the objective lens, we sealed the gap between the objective lens and the head bar with Blu-Tack (Bostik, UK). Images were acquired using commercial software (ScanImage 2016, Vidrio technologies, USA). In each trial, scanning began 1.2 s before the window opening and ended 0.8 s after closing.

### Analysis of behavioral performance

Unless stated otherwise, all data analyses were conducted with custom MATLAB codes (2019a, Mathworks, USA). Hit and correct rejection (CR) rates were calculated as the number of hits divided by the number of reward trials and the number of CRs divided by the number of no-reward trials, respectively. The correct rate was calculated as the number of hits and CRs divided by the total number of trials.

### Identifying high-performance periods

Periods of high behavioral performance were defined as follows. For each session, hit and CR rates were calculated in a sliding window of 50 trials. If both hit and CR rates were higher than 80%, then the trials in that window were included in the high-performance period. A high-performance session was defined as the one containing more than 200 trials in the high-performance period. All calcium data analyses were conducted for correct trials in high-performance periods of high-performance sessions except the SVM analysis in Fig. 2j–l for which all trials were used.

### Preprocessing calcium imaging data

Fluorescence signals (*F*) from individual regions of interest (ROIs) were extracted using the Suite2p package (python version)^68^. Before executing Suite2p, we manually inspected raw TIFF image stacks with ImageJ (NIH, USA) and excluded a few trials with significant motion artifacts (0.9% of all trials from 12 of 81 sessions in 6 of 24 mice). We smoothed raw fluorescence traces from each trial with a median filter (order 3) and concatenated them into a single trace for each detected ROI. Slow drifts in the fluorescence were corrected by subtracting the fifth percentile value within a sliding window of 900 frames. Baseline (*F*_*0*_) was determined as the mode of kernel density estimate (ksdensity function in MATLAB) of *F* in the entire trace. *ΔF*/*F*_*0*_ was calculated as (*F* - *F*_*0*_) / *F*_*0*_. Then, we determined a threshold for calcium events from *ΔF*/*F*_*0*_ values in the periods before the window opening as follows. First, we concatenated *ΔF/F*_*0*_ in the pre-window opening periods in all trials and found the three-interquartile value of *ΔF/F*_*0*_. To prevent large calcium activities from being included in this calculation, we excluded trials where any *ΔF/F*_*0*_ value in the pre-window opening period was found above the three-interquartile value. Then we calculated three-interquartile value again from the remaining trials and used it as a threshold for calcium events. We applied the threshold to the entire *ΔF/F*_*0*_ trace. Calcium event traces were obtained by preserving *ΔF/F*_*0*_ values above the threshold and replacing values below the threshold with zero. For most ROIs false positive ratio (number of negative events divided by number of positive events) was less than 5% (median=0%, interquartile range = 0.18%). We manually inspected ROIs with >5% false-positive ratio and found that the high false-positive ratios were due to a sparse calcium activity rather than an increased noise. Therefore, we did not further adjust the threshold for those ROIs to reduce the false-positive ratio.

To track the activity of the same neurons across days, we used publicly available cell registration software^69^. The open-source algorithm implemented probabilistic modeling of similarities between cells across multiple sessions. In detail, the spatial footprints similarities of neighboring cell pairs from different sessions were computed by using the centroid distances and spatial correlations, yielding estimated error rates (including false-positive and false-negative errors) of less than 5%. We adopted this algorithm while optimizing several parameters (e.g., 30° of maximal rotation, 12 μm of maximal distance to be considered as different cells).

### Neuronal responses to stimulus mice

Neuronal responses to each presented mouse were assessed by comparing areas under calcium events during the pre-stimulus (from the trial start to the window opening) versus the stimulus periods. Since it took about 400 ms for the window to completely open, the stimulus period was defined as the time interval between the completion of the window opening and the start of the response window.

### Discriminability index

We calculated the discriminability index (*d’*) to estimate the response preference of each neuron toward a trial type. *d’* was defined as follows:

1 $${d}^{{\prime} }=\,\frac{{\mu }_{1}-{\mu }_{2}}{\sqrt{\frac{1}{2}({\sigma }_{1}^{2}+\,{\sigma }_{2}^{2})}},$$

where *μ* and *σ* represent mean and standard deviations of calcium response amplitudes across trials of each type, respectively^70^.

When we analyzed temporal changes in the discriminability, we calculated *d’* values at each time point during the task. When we assessed if a neuron preferentially responded to the reward trials or no-reward trials, *d’* was calculated using mean calcium responses during the period between the window opening and the start of the response window. When we estimated if a neuron preferentially responded to a stimulus mouse in the four-mouse discrimination task, we calculated *d’* values separately for the reward and no-reward mice pairs. To estimate neuronal discriminability in the passive-viewing condition, we generated pairs of two mice from four social stimuli (i.e., a total of 6 pairs). We then averaged the *d’* values separately calculated for each pair. Mean calcium responses during the period between the opening and the closing of the interaction window were compared.

To test the significance of the *d’* value, we generated 1000 surrogate data sets in which trials were randomly permuted. If the experimentally-observed *d’* value fell within the top or bottom 2.5% of the distribution of the surrogate *d’*s, we considered it is trial-type specific. Therefore, 5% of neurons were expected to have a significant *d’* value in a given session by chance. For the passive presentation experiment, Bonferroni correction was applied to correct for multiple comparisons.

### Identification of reward-selective and mouse-selective neurons in reversal learning paradigms

If Go- or NoGo-preferring neurons maintained their preference after reversal learning, they were considered reward- or no-reward-selective neurons, respectively. Similarly, the neurons that reversed the Go- or NoGo-preference after reversal learning, and thus maintained the stimulus-mouse-preference were considered mouse-selective neurons. To test the statistical significance of the proportions of reward- and mouse-selective neurons, chi-squre tests were conducted. Specifically, for each reversed session pair, we constructed a 3 by 3 contingency table in which the column and row were the proportions of Go-, NoGo-preferring, and non-selective neurons on the day before and after the reversal, respectively. The null hypothesis was that Go-, NoGo-preferring, and non-selective neurons on day 1 (before the reversal) of the session pair were randomly assorted to Go-, NoGo-preferring, and non-selective neurons on day 2 (after the reversal). If the null hypothesis was rejected, post-hoc analyses were conducted to identify which proportions in the contingency table were significantly different from the chance by calculating adjusted residuals. Planned comparisons were made for the proportions of the neurons that maintained or reversed Go- and NoGo-preferrence across the reversal with Bonferroni correction (*p*-value criterion = 0.0125).

### Support vector machine (SVM) analysis

To test if neuronal population activity patterns provide task-relevant information, we used an SVM decoder with a linear kernel (fitcsvm in MATLAB with the standardization option) to classify neuronal activity patterns into either reward or no-reward trial categories (Figs. 2j–l and 6k, l). For each imaging session, independent SVMs were trained and tested at each time point in a leave-one-trial-out cross-validation procedure. Specifically, each decoder was trained with the neural population activity pattern from all trials except a withheld trial. Then we tested if the trained decoder classified the held-out trial into the correct category. For a session, the procedure was repeated by withholding a different trial at a time so that the withheld trials span the entire session. Decoding accuracy was expressed as the proportion of correct classifications.

For decoding mouse identity from the neural activity data obtained in the four-mouse discrimination tasks (Figs. 7g, h and 8g), we first down-sampled trials so that the number of trials for each stimulus mouse was the same. Then, we performed similar SVM analyses on reward and no-reward trials separately. For example, we trained and tested a decoder to classify neural activity patterns from reward trials into either one reward-associated mouse or another reward-associated mouse.

In the case of decoding individual-specific information in the passive-viewing condition (Fig. 7k, l), we implemented multiclass classification using SVM where binary classifiers distinguish between one (e.g., mouse A) and the rest (e.g., mouse B, C, and D). The classification of mouse type over four classifiers was done by a winner-takes-all strategy, thus allowing the chance level of 25%.

When we assessed the stability of individual mouse-specific information, we conducted SVM decoding analysis on each pair of task sessions imaged on different days. For each session pair, neurons detected on both days were included in the analysis. We trained an SVM decoder with the neuronal activity patterns in one session and tested the trained decoder on each trial in the other session (Fig. 8e, f).

To assess the statistical significance of the decoder performance, we performed a non-parametric cluster-based permutation test (1000 permutations)^71^.

### Statistics

Statistical differences between means were determined by unpaired *t*-test, chi-square test, Wilcoxon signed rank test or Kruskal–Wallis tests with post hoc Dunn’s multiple comparison tests, as mentioned in the text or figure legends. A *p*-value<0.05 was used as the criterion for statistical significance. All analyses were performed with MATLAB (2019a, Mathworks, USA). All data were expressed as mean±SEM.

### Reporting summary

Further information on research design is available in the Nature Portfolio Reporting Summary linked to this article.

## Supplementary information

Supplementary Information

Reporting Summary

## Data Availability

Data are available upon request to the corresponding author. Source data are provided with this paper.

## Source data

Source Data

## Code availability

Customized code to reproduce the figures is available upon request to the corresponding author.
