# Supplementary material for: Dynamic and stable hippocampal representations of social identity and reward expectation support associative social memory in male mice
Source: Nat Commun. 2023 May 5;14:2597. doi: 10.1038/s41467-023-38338-3 (PMC10163237; doi:10.1038/s41467-023-38338-3)
Supplement: Supplementary file 1 — Supplementary Information [file 41467_2023_38338_MOESM1_ESM.pdf]

Dynamic and stable hippocampal representations of social identity and reward expectation support associative social memory in male mice

Authors: Eunji Kong[1,2,3,#], Kyu-Hee Lee[1,#], Jongrok Do[1,4], Pilhan Kim[2,3*], Doyun Lee[1,*]

Affiliations

[1]Center for Cognition and Sociality, Institute for Basic Science, Daejeon, 34126, Republic of Korea.

[2]Graduate School of Medical Science and Engineering, Korea Advanced Institute of Science and Technology, Daejeon, 34141, Republic of Korea.

[3]KI for Health Science and Technology (KIHST), Korea Advanced Institute of Science and Technology, Daejeon, 34141, Republic of Korea.

[4]Department of Biological Sciences, Korea Advanced Institute of Science and Technology, Daejeon, 34141, Republic of Korea.

[#]These authors contributed equally: Eunji Kong, Kyu-Hee Lee

*Correspondence to: Doyun Lee (leedoyun@ibs.re.kr) or Pilhan Kim (pilhan.kim@kaist.ac.kr)

This file includes Supplementary Figures 1-11.

Supplementary Figure 1

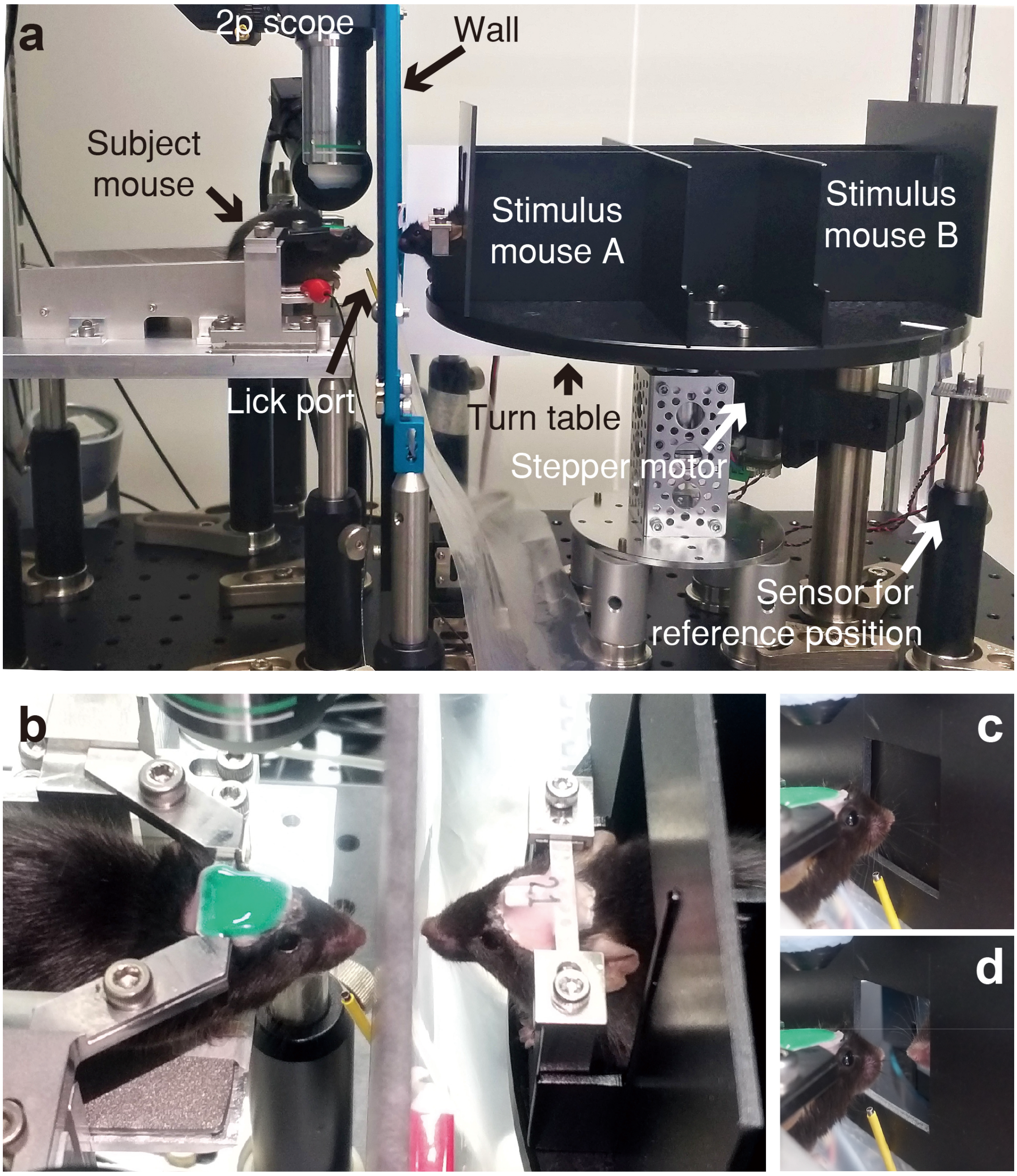

Supplementary Fig. 1. Behavioral setup for individual discrimination. a Arrangement of the parts and devices used for individual discrimination tasks. **b** Picture showing the distance between a subject and a stimulus mouse at the interaction position. **c, d** Interaction window in the closed (**c**) and open (**d**) states.

Supplementary Figure 2

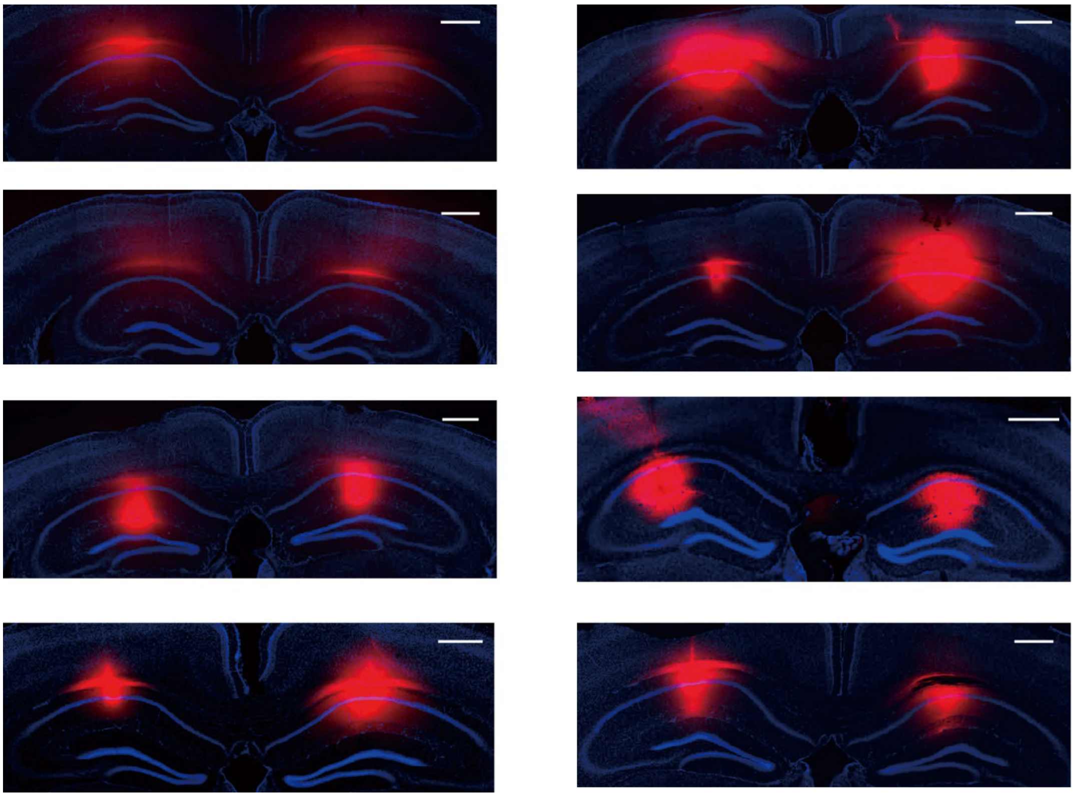

Supplementary Fig. 2. Images showing spread of fluorescent muscimol in the dorsal hippocampus of the mice used in Fig. 2c-f. Red, BODIPY-conjugated muscimol. Blue, DAPI. Scale bar, 500 μm.

Supplementary Figure 3

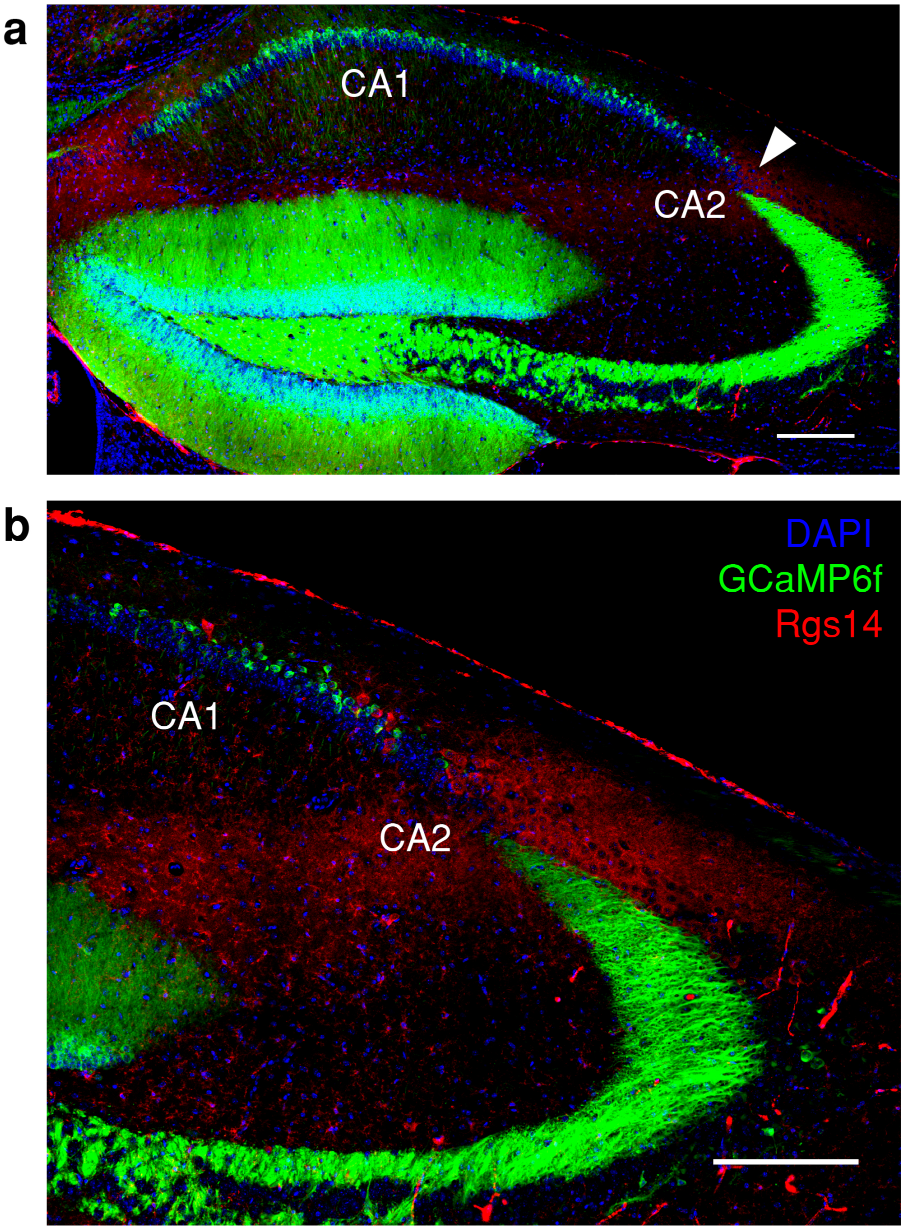

Supplementary Fig. 3. Differential expression of GCaMP6f in dorsal hippocampal subregions in Thy1-GCaMP6f transgenic mice. a GCaMP6f signals are enriched in CA1 neurons, dentate granule cells, and moss fiber tract. **b** Magnified image around the area indicated with white arrowhead in (**a**). GCaMP6f signals are not detectable in Rgs14-positive CA2 neurons and CA3 neurons. Therefore, we selectively monitored neural activities of dorsal CA1 neurons, but not CA2 neurons. Blue, DAPI. Green, GCaMP6f. Red, Rgs14. Scale bar, 200 µm. Similar results were observed in two different mice.

Supplementary Figure 4

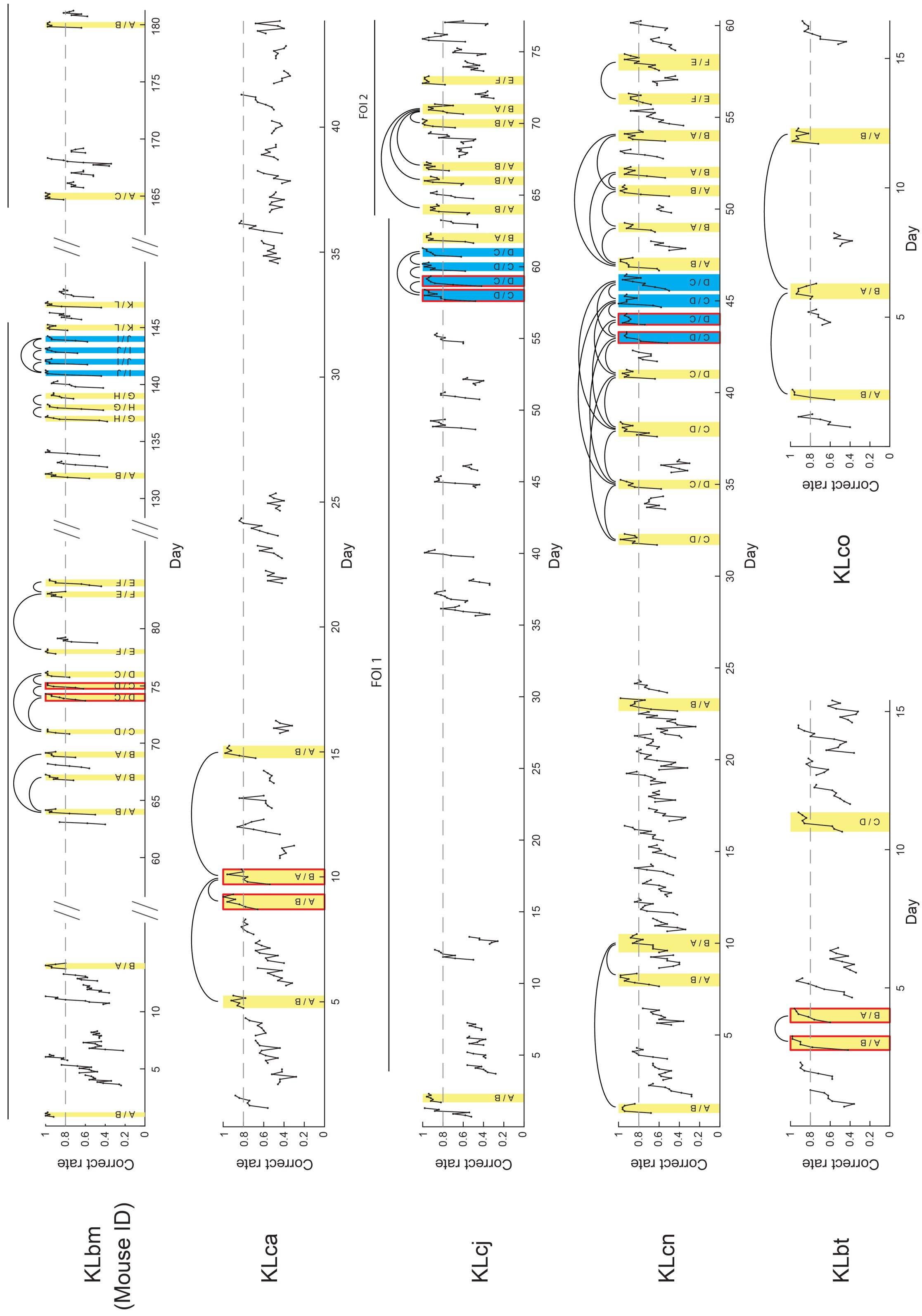

Supplementary Fig. 4. Identification of session pairs with reversed stimulus reward contingency. Each panel shows the behavioral performance of all imaging sessions for each subject mouse. Each black dot represents the mean correct rate calculated for fifty trials. All these imaging sessions and additional imaging sessions from five other mice that failed to learn the reversed rule were included for the SVM analysis in Fig. 2l and supplementary Fig. 5. Colored bars indicate high performance sessions. IDs of reward- and no-reward-associated mice in the bar. Each pair of sessions with reversed stimulus-reward contingency are marked with a curved line. All these pairs comprise the dataset used for Supplementary Fig. 8. The five pairs of the bars with red edges represent the session pairs for which reversal learning was done in one day. These pairs were used for analyses in Fig. 3. The three groups of blue bars are four consecutive sessions in which reversal learning was achieved daily. These three groups of sessions were used for the analyses in Fig. 4a-c. For the mouse KLbm, calcium imaging was done in two different fields, which are marked by the lines above the panel. Source data are provided as a Source Data file.

Supplementary Figure 5

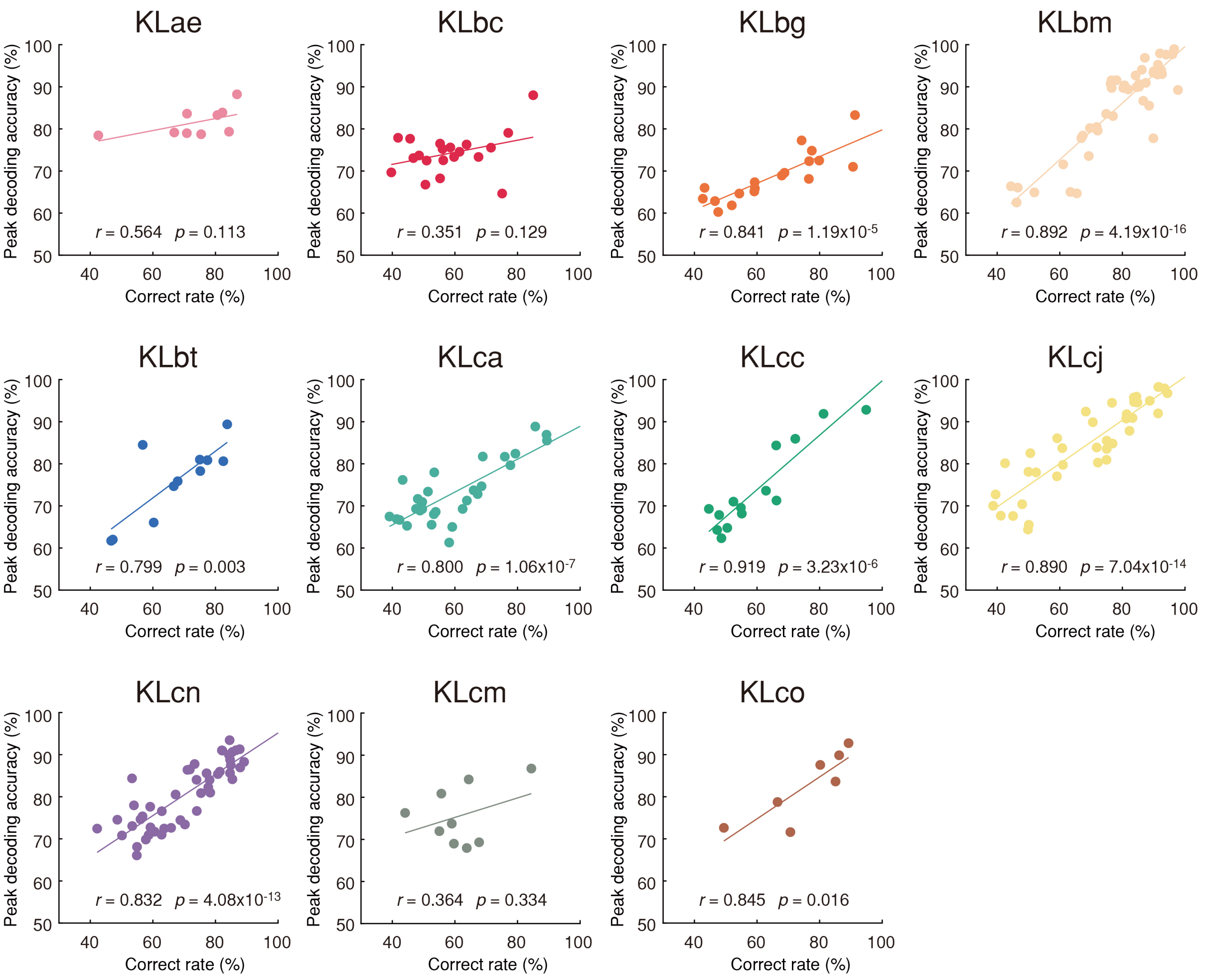

Supplementary Fig. 5. Correlation between SVM decoding accuracy and behavioral performance. For each imaging session included in Fig. 2l, SVM decoding accuracy was calculated, and the peak accuracy was plotted against behavioral performance. Each plot corresponds to a different mouse. The correlation (Pearson's correlation) between the SVM decoding accuracy and the behavioral performance was significant in 8 mice ($p < 0.05$). Mouse identification is shown above each plot. Source data are provided as a Source Data file.

Supplementary Figure 6

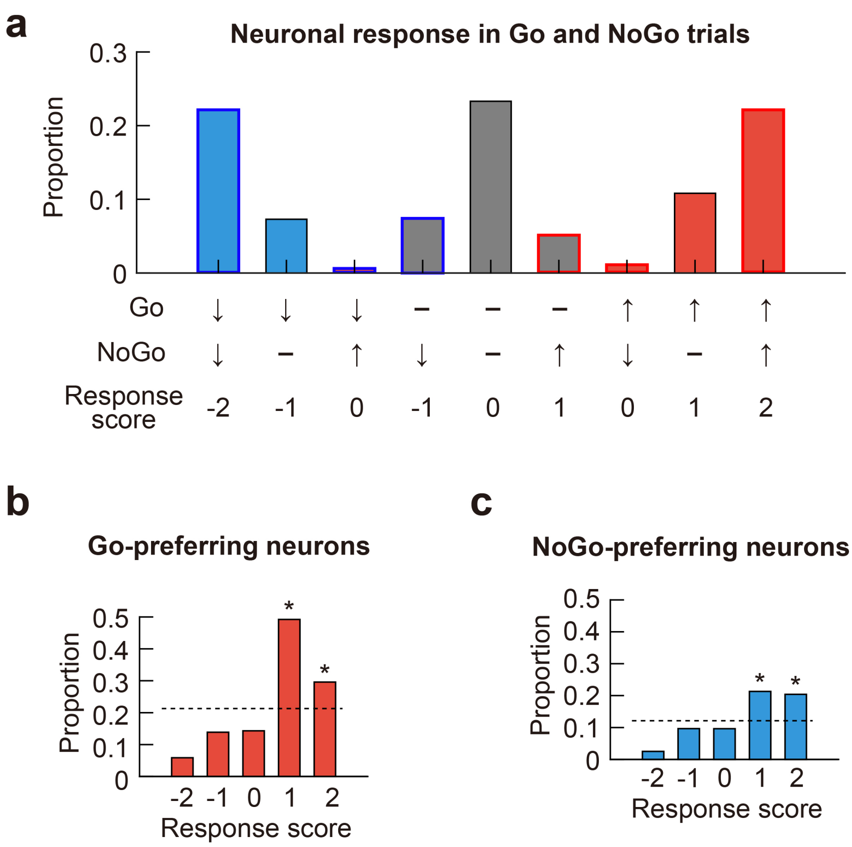

Supplementary Fig. 6. Neuronal responsivity in Go and NoGo trials affects trial type preference of dorsal CA1 neurons. a Proportions of the neurons that displayed the same or different responses in Go and NoGo trials. Scores of 1, 0, -1 for activation, no response, and inhibition. **b, c** Proportions of Go-preferring (**b**) and NoGo-preferring (**c**) neurons among the neurons with each response score on day 1. The broken lines indicate the chance to be Go-preferring (**b**) and NoGo-preferring (**c**) on day 1. The chance levels equal to the proportions of Go- and NoGo-preferring neurons on day 1 in Fig. 3h. A 3 by 5 contingency matrix (three different neuronal preferences, Go, NoGo and none; five levels of response scores) was constructed and chi-square test was performed on it ($p = 1.2 \times 10^{-68)}$. A post-hoc analysis was conducted on adjusted residuals with Bonferroni correction to find which proportions were significantly higher than the chance ($p = 4.3 \times 10^{-33}$ and 7.4×10^{-6} for response score of 1 and 2 in (**b**), $p = 7.7 \times 10^{-7}$ and 6.6×10^{-8} for response score of 1 and 2 in (**c**)). For the post-hoc test, a planned comparison was made for Go and NoGo-preferring neurons with each response score. Source data are provided as a Source Data file.

Supplementary Figure 7

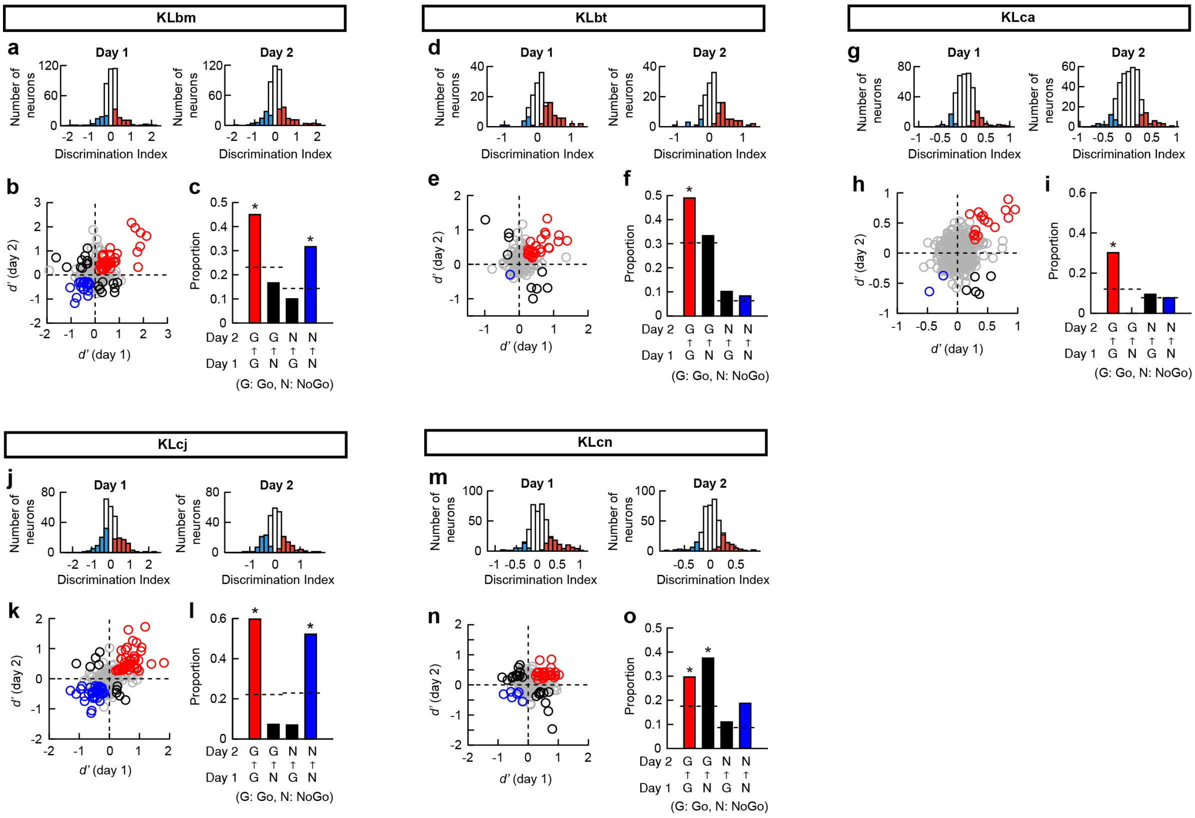

Supplementary Fig. 7. Neuronal response selectivity in each subject mouse in Fig. 3. a, d, g, j, m Distribution of *d'* and significant *d'* values (colored bars, shuffling test, two-sided, $p < 0.05$). **b, e, h, k, n** Reward- (red) and no-reward-selective (blue) neurons that maintained Go- and NoGo-preference during reversal learning, respectively. Circles indicate individual neurons. Black circles indicate the neurons changed their preference. **c, f, i, l, o** Proportions of reward- (*red*) and no-reward-selective (*blue*) neurons are higher than chance (chi-square test followed by a post-hoc test analysis on adjusted residuals with Bonferroni correction; KLbm: $p = 1.0 \times 10^{-7}$ for G → G, $p = 1.6 \times 10^{-5}$ for N → N; KLbt: $p = 3.3 \times 10^{-4}$ for G → G; KLca: $p = 5.8 \times 10^{-6}$ for G → G; KLcj: $p = 1.6 \times 10^{-19}$ for G → G, $p = 9.7 \times 10^{-12}$ for N → N; KLcn: $p = 2.9 \times 10^{-4}$ for G → G, $p = 0.001$ for N → G). The broken lines indicate the chance to be Go- or NoGo-preferring on day 2. The chance levels are equal to the proportions of Go- and NoGo-preferring neurons on day 2 in (**a**, **d**, **g**, **j**, and **m**). Source data are provided as a Source Data file.

Supplementary Figure 8

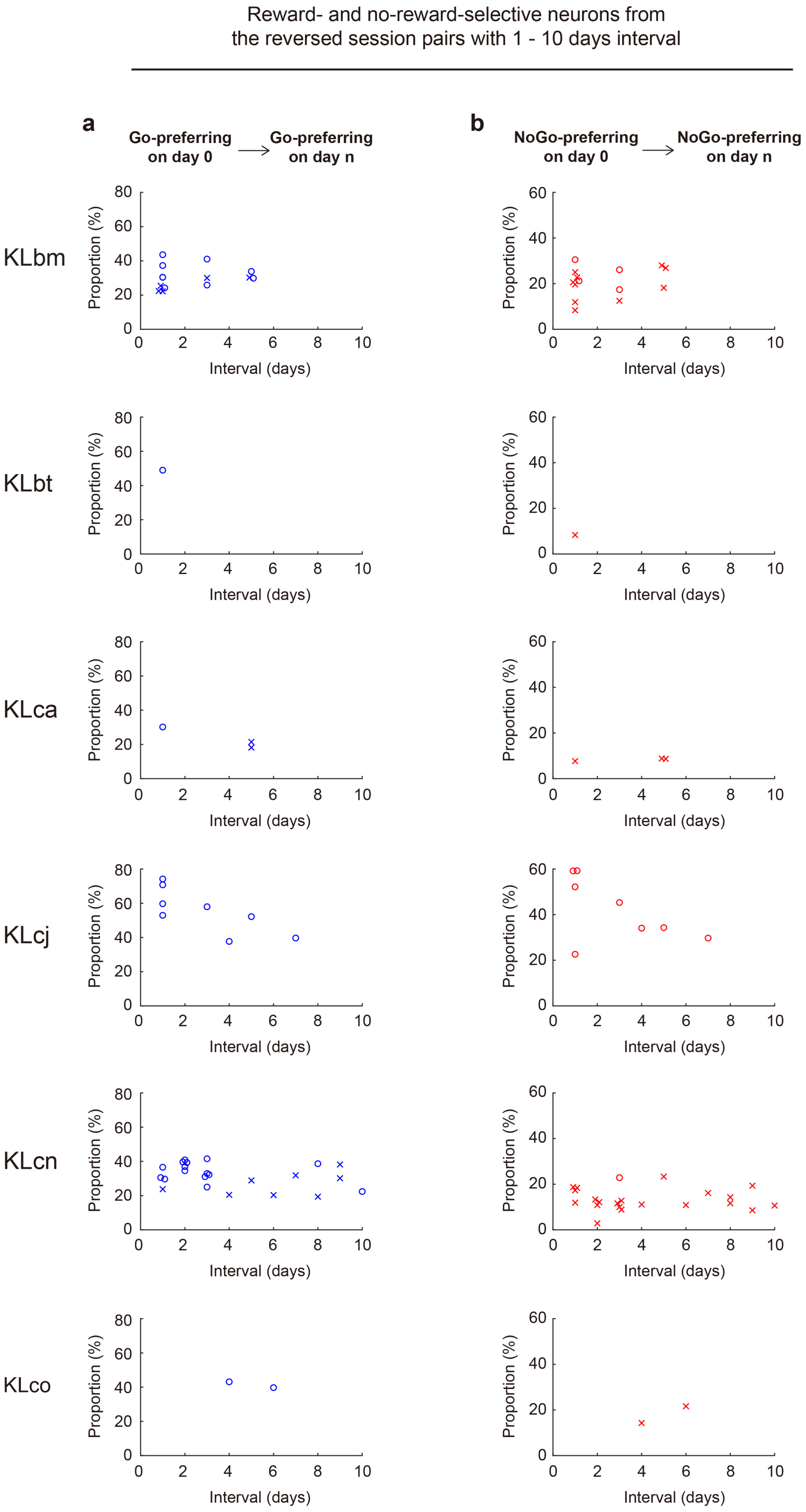

Supplementary Fig. 8. Reward- and no-reward-specific neurons identified from the session pairs up to 10 days apart. a and **b** Proportions of Go- (**a**) or NoGo-preferring (**b**) neurons on the day after the reversal among the Go- (**a**) or NoGo-preferring (**b**) neurons on the day before the reversal. Open circles indicate the statistically significant ($p < 0.05$) proportions while crosses indicate the proportions that did not pass the statistical test (Chi-square test followed by post-hoc analysis on adjusted residuals with Bonferroni correction). Source data are provided as a Source Data file.

Supplementary Figure 9

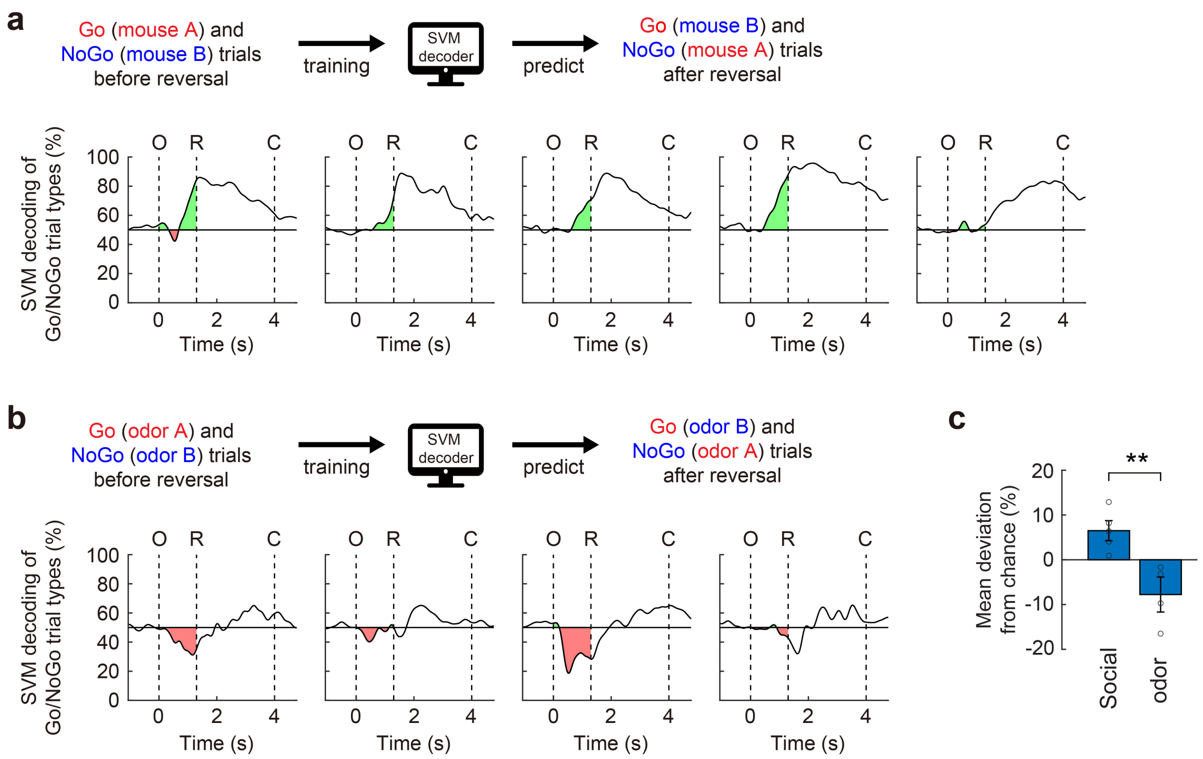

Supplementary Fig. 9. Population activity in the dorsal CA1 better predicts Go/NoGo trial types during social than non-social task. a, b Top: a schematic of SVM decoding. At each time point independent SVM decoders were trained with the population activity pattern on the day before the reversal learning and tested on the population activity on the day after the reversal. Bottom: SVM decoding accuracies for Go/NoGo trials types for the social (**a**) and non-social task (**b**). Note that decoding accuracies below 50% indicate that SVM decoder better predicted stimuli than Go/NoGo trial types. **c** Comparision of mean SVM decoding accuracies in the O-R period between the social and non-social odor discrimination tasks (mean ± SEM, two-sided unpaired t test, ** p = 0.0067, five and four mice for social and non-social tasks). Source data are provided as a Source Data file.

Supplementary Figure 10

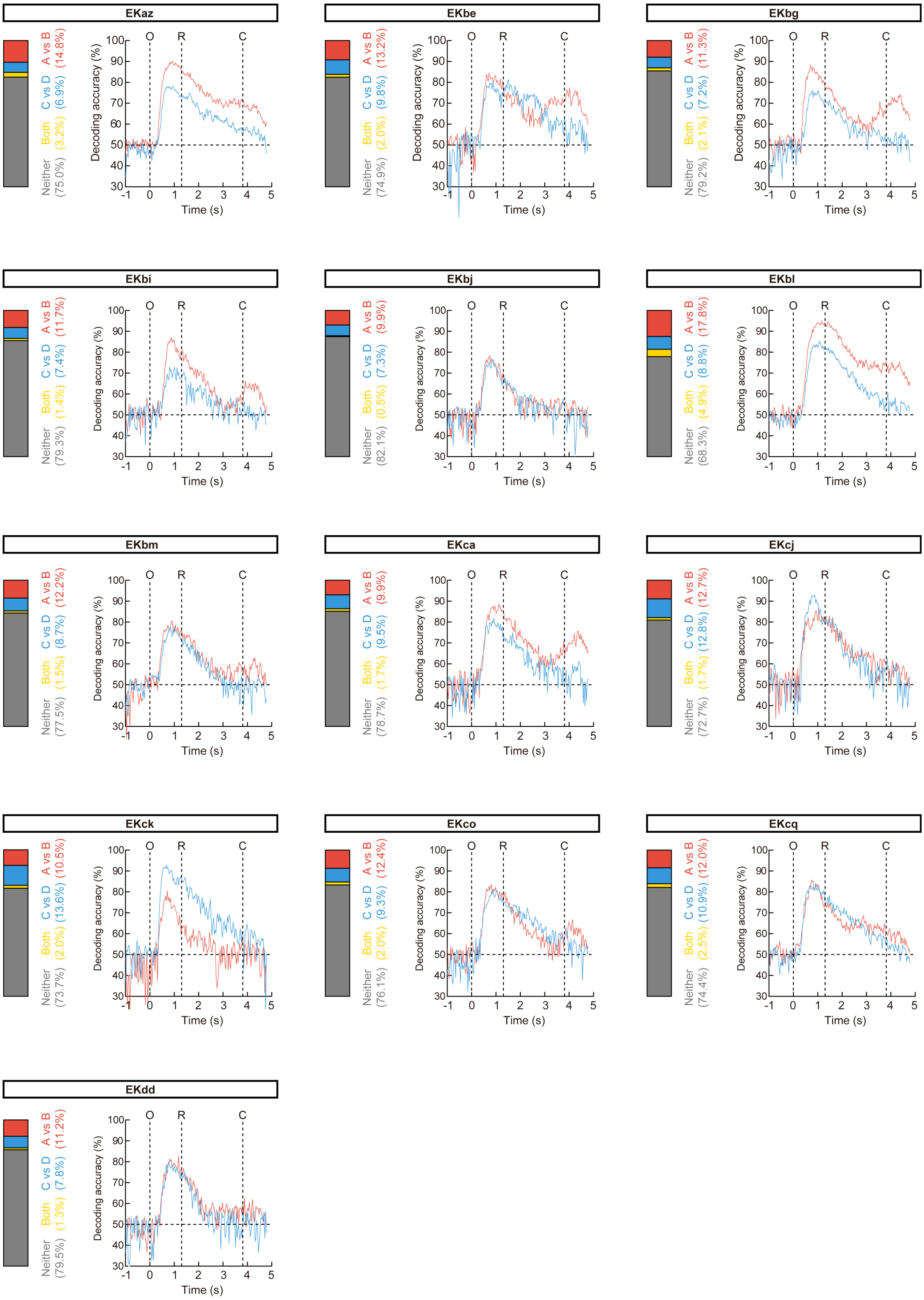

Supplementary Fig. 10. Hippocampal neurons encode social identity at both single-cell and population levels. Each panel displays data from each subject mouse included in Fig. 7d, g. Left, Proportions of neurons that distinguish between the two stimulus mice in the reward (*red*), no-reward (*blue*), and both categories (*yellow*). Gray indicates the proportion of non-discriminating neurons. Right, SVM decoding accuracies of stimulus mice in the reward (*red*) and no-reward (*blue*) category. Separate SVM decoders were trained and tested at each time point (leave-one-out cross-validation). Vertical dashed lines indicate the window opening (O), start of response window (R), and window closing (C). For all panels, mean ± SEM (shaded area) is presented. Source data are provided as a Source Data file.

Supplementary Figure 11

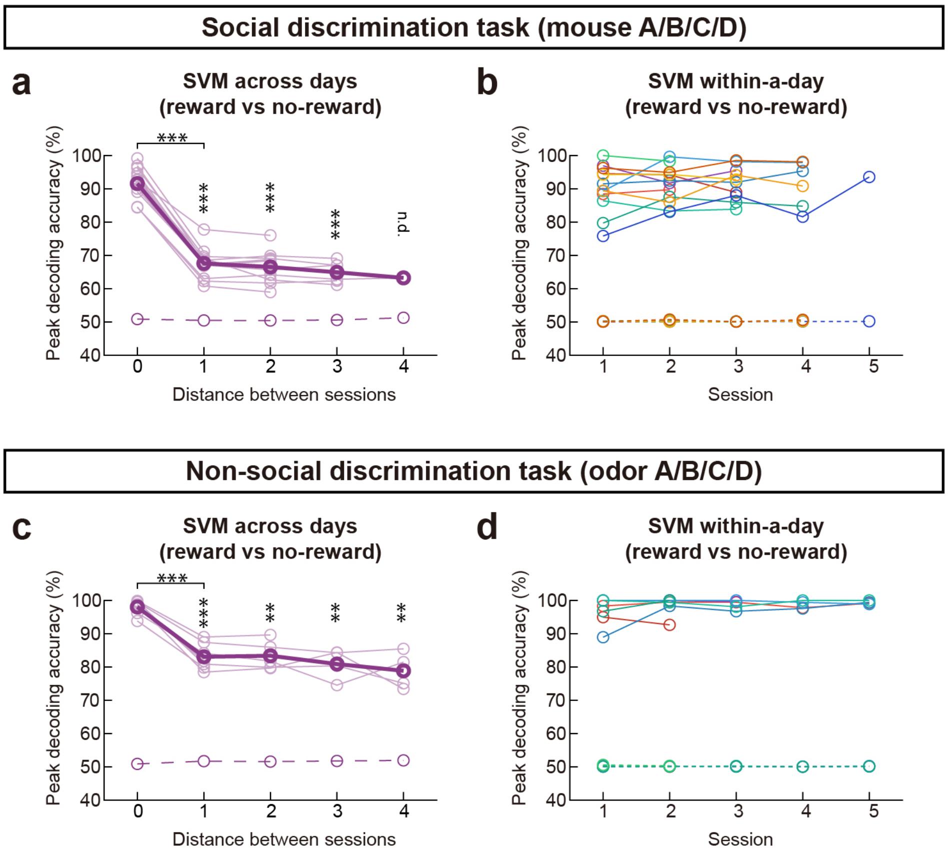

Supplementary Fig. 11. Dynamic neural representation of trial types (Go or NoGo) during four-mouse discrimination and four-odor discrimination tasks. **a, c** Similar to Fig. 8f and Fig. 9h, decoding accuracies of trial types decreased as the time interval between sessions increased. The SVM decoders trained with neuronal activity patterns on day 1 were tested on each trial of day n (2 to 5). For the zero distance, within-day decoding accuracy on day 1 was calculated. Across-day decoding accuracies were maintained higher than chance level (two-sided Wilcoxon signed-rank test; social task (a): $p = 1.0 \times 10^{-5}$, 1.8×10^{-5}, 1.1×10^{-4} for distance 1, 2, 3, $p = 3.7 \times 10^{-5}$ for 0 vs. 1; non-social task (c): $p = 5.8 \times 10^{-4}$, 0.003, 0.006, 0.006 for distance 1, 2, 3, 4, $p = 5.8 \times 10^{-4}$ for 0 vs. 1). n.d., not determined. **b, d** Similar to Fig. 8g and Fig. 9i, decoding accuracies of trial types maintained high when the SVM decoder was trained and tested within each session. Each color indicates each subject mouse and dashed lines represent peak decoding accuracies obtained from 1000 shuffled data. Source data are provided as a Source Data file.
