# Supplementary material for: Dynamic and stable hippocampal representations of social identity and reward expectation support associative social memory in male mice
Source: Nat Commun. 2023 May 5;14:2597. doi: 10.1038/s41467-023-38338-3 (PMC10163237; doi:10.1038/s41467-023-38338-3)
Supplement: Supplementary file 2 — Reporting Summary [file 41467_2023_38338_MOESM2_ESM.pdf]

nature portfolio

Corresponding author(s): Doyun Lee

Last updated by author(s): Mar 21, 2023

Reporting Summary

Nature Portfolio wishes to improve the reproducibility of the work that we publish. This form provides structure for consistency and transparency in reporting. For further information on Nature Portfolio policies, see our Editorial Policies and the Editorial Policy Checklist.

Statistics

For all statistical analyses, confirm that the following items are present in the figure legend, table legend, main text, or Methods section.

n/a	Confirmed	
☐	☒	The exact sample size (*n*) for each experimental group/condition, given as a discrete number and unit of measurement
☐	☒	A statement on whether measurements were taken from distinct samples or whether the same sample was measured repeatedly
☐	☒	The statistical test(s) used AND whether they are one- or two-sided<br>*Only common tests should be described solely by name; describe more complex techniques in the Methods section.*
☒	☐	A description of all covariates tested
☐	☒	A description of any assumptions or corrections, such as tests of normality and adjustment for multiple comparisons
☐	☒	A full description of the statistical parameters including central tendency (e.g. means) or other basic estimates (e.g. regression coefficient) AND variation (e.g. standard deviation) or associated estimates of uncertainty (e.g. confidence intervals)
☐	☒	For null hypothesis testing, the test statistic (e.g. *F*, *t*, *r*) with confidence intervals, effect sizes, degrees of freedom and *P* value noted<br>*Give P values as exact values whenever suitable.*
☒	☐	For Bayesian analysis, information on the choice of priors and Markov chain Monte Carlo settings
☒	☐	For hierarchical and complex designs, identification of the appropriate level for tests and full reporting of outcomes
☐	☒	Estimates of effect sizes (e.g. Cohen's *d*, Pearson's *r*), indicating how they were calculated

Our web collection on statistics for biologists contains articles on many of the points above.

Software and code

Policy information about availability of computer code

Data collection	Behavioral data were recorded using custom-written software in MegunoLink Pro (Number Eight Innovation) and Arduino IDE 1.8.3 (open source). Calcium imaging data were recorded using ScanImage 2016b (Vidrio Technologies).
Data analysis	For calcium data, ROIs were detected using Suite2p (open source). All other data analysis was conducted using custom-written MATLAB (2019b) codes.

For manuscripts utilizing custom algorithms or software that are central to the research but not yet described in published literature, software must be made available to editors and reviewers. We strongly encourage code deposition in a community repository (e.g. GitHub). See the Nature Portfolio guidelines for submitting code & software for further information.

Data

Policy information about availability of data

All manuscripts must include a data availability statement. This statement should provide the following information, where applicable:

- Accession codes, unique identifiers, or web links for publicly available datasets
- A description of any restrictions on data availability
- For clinical datasets or third party data, please ensure that the statement adheres to our policy

Data are available upon request to the corresponding author. Source data are provided with this paper.

Human research participants

Policy information about studies involving human research participants and Sex and Gender in Research.

Reporting on sex and gender	not applicable
Population characteristics	not applicable
Recruitment	not applicable
Ethics oversight	not applicable

Note that full information on the approval of the study protocol must also be provided in the manuscript.

Field-specific reporting

Please select the one below that is the best fit for your research. If you are not sure, read the appropriate sections before making your selection.

☒ Life sciences ☐ Behavioural & social sciences ☐ Ecological, evolutionary & environmental sciences

For a reference copy of the document with all sections, see nature.com/documents/nr-reporting-summary-flat.pdf

Life sciences study design

All studies must disclose on these points even when the disclosure is negative.

Sample size	No statistical method was used to predetermine sample size. Our sample sizes were chosen to be similar to accepted standards in the field.
Data exclusions	No data was excluded from the analysis
Replication	All experiments were independently conducted for each subject mouse, and all results were pooled.
Randomization	Within-subject comparisons were made in most experiments. Mice were randomly assigned to the experimental or control group when necessary.
Blinding	Trial types were randomly determined by a custom-written code. All data analysis was performed by running the same analysis code on each trial type, so they did not require blinding.

Reporting for specific materials, systems and methods

We require information from authors about some types of materials, experimental systems and methods used in many studies. Here, indicate whether each material, system or method listed is relevant to your study. If you are not sure if a list item applies to your research, read the appropriate section before selecting a response.

Materials & experimental systems

n/a	Involved in the study	
☐	☒	Antibodies
☒	☐	Eukaryotic cell lines
☒	☐	Palaeontology and archaeology
☐	☒	Animals and other organisms
☒	☐	Clinical data
☒	☐	Dual use research of concern

Methods

n/a	Involved in the study	
☒	☐	ChIP-seq
☒	☐	Flow cytometry
☒	☐	MRI-based neuroimaging

Antibodies

Antibodies used	1) mouse anti-Rgs14 (Neuromabs, 75-170), 2) goat anti-mouse Alexa 647 antibody (Abcam, ab150115)
Validation	Quality control information and relevant citations for antibodies used in this study are available at manufacturer's website as following:<br>1) mouse anti-Rgs14 - https://neuromab.ucdavis.edu/datasheet/N133_21.pdf<br>2) goat anti-mouse Alexa 647 antibody - https://www.abcam.com/goat-mouse-igg-hl-alexa-fluor-647-ab150115.html

Animals and other research organisms

Policy information about studies involving animals; ARRIVE guidelines recommended for reporting animal research, and Sex and Gender in Research

Laboratory animals	2-11 months old male C57BL/6J mice.<br>2-10 month-old male Thy1-GCaMP6f mice.<br>After surgery, pairs of stimulus mice used in the two-mouse discrimination task were housed separately in the same cage partitiond with a transparent and multiperforated wall. All other mice were housed individually.<br>The mice were maintained on a 12 h: 12 h ligh:dark cycle at 22 ˚C with 50% humidity.
Wild animals	The study did not involve wild animals.
Reporting on sex	Only male mice were used in the study.
Field-collected samples	The study did not involve samples collected from the field.
Ethics oversight	All procedures used in this work were approved by the Institutional Animal Care and Use Committee of the Institute for Basic Science.

Note that full information on the approval of the study protocol must also be provided in the manuscript.
